# Impact of sodium doping on the structural properties of CQDs

**DOI:** 10.1038/s41598-025-24609-0

**Published:** 2025-11-19

**Authors:** Pawan Kumar, Shweta Dua, M. Sridevi, Karsimran Singh, Rajiv K. Singh, Manoj Kumar Khanna, Ravinder Kaur, Geeta Bhatt

**Affiliations:** 1 Ramjas College, University of Delhi, New Delhi, 110007 India; 2 Department of Electronic Science, South Campus University of Delhi, New Delhi, 110021 India; 3 Bhaskarcaharya College of Applied Sciences, University of Delhi, New Delhi, 110075 India; 4 Photovoltaic Metrology Section, Advanced Materials & Device Metrology Division, CSIR-National Physical Laboratory, Dr K. S. Krishnan Marg, New Delhi, 110012 India; 5 Department of Physics and Astrophysics, University of Delhi, New Delhi, 110007 India; 6 Deen Dayal Upadhyaya College, University of Delhi, New Delhi, 110075 India; 7 Non-Collegiate Women’s Education Board, University of Delhi, New Delhi, 110007 India

**Keywords:** Carbon quantum dots (CQDs), EDTA, Thermal stability, Chemistry, Materials science, Nanoscience and technology

## Abstract

Nitrogen- and sodium-doped carbon quantum dots (CQDs) were synthesized from citric acid monohydrate and urea, using varying concentrations of Ethylenediaminetetraacetic acid disodium salt dehydrate (EDTA) in a bottom-up approach with microwave irradiation. First, a comparative study of synthesis duration and EDTA concentration was conducted. Upon optimization, our results showed that E1-CQD@300sec exhibited the highest absorbance among all EDTA-CQD samples, with absorbance values following this trend: EDTA-CQD@300sec > EDTA-CQD@225sec > EDTA-CQD@165sec for most EDTA concentrations. Additionally, photoluminescence (PL) was found to follow the order: E2-CQD@165sec > E1-CQD@225sec ≈ E1-CQD@300sec. E1-CQD@300sec had a spherical morphology with an approximate size of 13 nm, as observed in high-resolution transmission electron microscopy (HR-TEM). This E1-CQD@300sec sample has a bandgap of 1.95 eV, with a highest occupied molecular orbital (HOMO) of -5.15 eV and a lowest unoccupied molecular orbital (LUMO) of -3.2 eV. X-ray diffraction (XRD) revealed a broad peak at ~ 26.45°, indicating an amorphous structure, which was confirmed by Raman spectroscopy, showing an I_D_/I_G_ ratio of 1.11. FT-IR analysis indicated the presence of nitrogen-based functional groups, such as C-N and N-H, on the surface. With increase in EDTA concentration, the bandgap of EDTA-CQD@300sec increased, reaching a maximum of 2.29 eV for E5-CQD@300sec, with a HOMO of -5.55 eV and a LUMO of -3.26 eV, and other functional groups, such as C ≡ C and C ≡ N, were also detected in the FT-IR spectra. In addition, agglomeration was further increased in the presence of EDTA, possibly due to uncontrolled nucleation, EDTA-CQD complex formation, high EDTA concentration, and insufficient surface coverage by EDTA, as observed through dynamic light scattering (DLS). The thermal stability of ETDA-CQD@300sec also found to decreased from ~  208°C to 40 °C. Further modifications and optimization is necessary for potential use of synthesized CQDs in the opto-electronic applications.

**Supplementary Information:**

The online version contains supplementary material available at 10.1038/s41598-025-24609-0.

## Introduction

Carbon quantum dots (CQDs), a novel type of carbon nanomaterials and smaller than 10 nm^1^ are bright photoluminescent quasi-spherical nanoparticles with amorphous to nanocrystal cores. CQDs have a self-passivating shell made up of functional groups and a carbon core that is 2–3 nm in size with a 0.2 nm lattice spacing^2^. Investigations have shown that the core (intrinsic states) can be amorphous (mixed sp^2^/sp^3^) or graphitic crystalline (sp^2^)^3,4^. Structure of CQDs depends on various factors like precursor, solvent, synthesis method, solution pH, synthesis duration, etc^5^.

In 2004, Xu. and his colleagues inadvertently discovered fluorescent nanoparticle while purification of single-walled carbon nanotubes (SWCNTs) which was later named as CQDs. This encouraged the scientists to investigate the fluorescence properties of CQDs and develop an entirely new class of fluorescent carbon nanomaterial. Two years later, in 2006 Sun et al. successfully established a synthetic method for producing CQDs with intense fluorescence emission through surface passivation. With passage of time, different techniques were developed for the CQDs synthesis which are broadly categorized into top-down or bottom-up approach. The top-down approach essentially involves the destruction of carbonaceous macromolecules into nano-sized CQDs by chemical, physical, or electrochemical methods. On contrary, bottom-up approach is based on the synthesis of the nanostructures from organic molecular precursors involving series of polymerization and carbonisation reactions^6^.

CQDs are inexpensive and possess various fascinating qualities like tunable excitation-emission, outstanding catalytic properties, high photo-stability, high photo-response, good water solubility, and easy surface functionalization^7–10^. These unique qualities have made CQDs popular in a variety of applications, including photovoltaics, optronics, single-electron transistors, medical diagnostics, sensing, drug delivery, catalysts, and photocatalysis^11–14^. In addition, CQDs being ecofriendly and biocompatible have gained great deal of interest in bio-sensing, bio-medical and bio-imaging applications where conventional semiconductor quantum dots (SQDs) and organic dyes can’t be employed as they are hazardous due to the presence of heavy metals despite possessing excellent fluorescence properties^6^. Thus, CQDs have emerged as rising stars among other carbon-based nanoparticles and are now regarded as an incredibly valuable asset of nanotechnology^15^.

The fascinating optical properties of CQDs can be easily altered by various means (i) doping/co-doping with heteroatom and (ii) Surface engineering Surface passivation/surface functionalization^16^. Doping modifies the structural and chemical composition of CQDs, and thus their optical, electrical, and chemical properties. Consequently, it expands the range of their application domains^17^. Furthermore, studies have shown that doping atoms greatly increase the fluorescence quantum yield of CQDs^18^. Nitrogen has an atomic size comparable to that of carbon with five coordinating electrons. Thus, it has the ability to replace carbon atoms or form chemical bonds with them, changing the surface characteristics and electronic structure of CQDs. As per reports, nitrogen doping increases the degree of conjugation and electron density of CQDs, and, thus their quantum yield. It also modifies the wavelength at which fluorescence emission occurs, and enhances both selectivity and stability^17^. Nitrogen rich CQDs with donor-π-acceptor (D-π-A) configurations are interesting because they promote rigid planes and increase crystallinity^19^. In addition, the doping has ability to enhance the conductivity along with the optical properties of the CQDs. Other than Nitrogen various dopants have been investigated like boron (B), fluorine (F), Sulphur (S), phosphorous (P)^20^ but doping of metal ion has an added advantage over them due to the donor nature of metal ions that makes it an effective method for producing highly-efficient luminescent CQDs^21–23^. Up to now, a variety of metal ions, such as Zn^2+^, Mn^2+^, K^+^, and Cu^2+^, have been used to enhance the PL of CQDs^23–26^ and very few have reported the impact of sodium doping (Na)^26–28^. Since, Sodium has lowest electronegativity (0.93) amongst Carbon (2.55), Nitrogen (3.04) and Oxygen (3.44) so it can easily contribute to the conductivity.

Surface engineering have been reported with variety of surface passivating agents like linear structured polyethyleneimine (LPEI), oligomers (poly ethylene glycol (PEG)), thionyl chloride, thiols and spiropyrans, etc. These agents create a thin protective layer that prevents impurities from adhering to carbon quantum dots (CQDs), enhancing their stability and longevity. This layer also prevents the loss of photo-induced carriers from surface sites, which in turn improves the fluorescence intensity of the CQDs. Compared to bare CQDs, those with surface passivation exhibit significantly higher optical activity, showing strong photoluminescence (PL) across visible to near-infrared regions. Surface passivation boosts the quantum yield of CQDs to a maximum of 55–60%^1,29^.

Usually, doping and passivation are done separately but here doping with passivation has been approached in a single step. This treatment not only reduces the number of steps but also the make it very green and scalable. To achieve this, EDTA was chosen due to the following reasons: (1) low toxicity, (2) water soluble, (3) ease of availability, and (4) improves solubility of CQDs in water, (5) affordable and (6) functionalizes CQDs’ surface as well^30–32^, .

In the present work we report the synthesis of CQDs, its confirmation and the effect of EDTA on the structural properties of CQDs. In the experiment, the distilled water was used as solvent, citric acid monohydrate and Urea as precursors, and EDTA as the passivating and doping agent. EDTA-CQDs synthesis was carried out using microwave oven for three different times: 165 s, 225 s and 300 s. At each of these times, the concentration of EDTA was varied from 0.0 g to 1.0 g in steps of 0.2 g. This approach allowed us to optimize the synthesis time for EDTA-CQDs, which exhibit excellent optical properties that, to the best of our knowledge, have not been reported before. Initially, we discuss the structural properties of the CQD@300sec, including absorbance, photoluminescence (PL), Raman spectroscopy, X-ray diffraction (XRD), and high-resolution transmission electron microscopy (HR-TEM). This is followed by the optimization of the EDTA-CQDs synthesis time based on the best optical performance. Finally, we carried out various investigations on all the EDTA-CQDs samples synthesized at 300 s, including bandgap measurement, thermal stability, crystallinity, size distribution, cyclic voltammetry, and FTIR analysis.

## Materials and methods

### Materials

Citric acid monohydrate (C_6_H_8_O_7_.H_2_O, 99.5%) were procured from CDH Fine chemicals and urea (CH_4_N_2_O, 99.5%) as well as Ethylenediaminetetraacetic acid disodium salt dehydrate (C_10_H_14_N_2_O_8_Na_2_.2H_2_O, 99%) abbreviated as EDTA from HiMedia laboratories Pvt. Ltd. Dimethylformamide (DMF), Acetone (99.5%), Iso-propyl alcohol (99.5%), Tetrabutylammonium hexafluorophosphate (TBAPF_6_) was purchased from sigma Aldrich. All reagents were of at least analytical grade purity and were used as it is without further purification.

Glassware used in all the experiments were thoroughly cleaned using soap solution, distilled water and acetone before use and the entire experiment was carried under the ambient conditions.

### Methods

#### Synthesis of CQDs

A solution of 1 g of CA and Urea was prepared in 20 ml distilled water and 06 such beakers were prepared. Then, different concentration of EDTA (0.0 g, 0.2 g, 0.4 g, 0.6 g, 0.8 g and 1.0 g) was added in each of the above prepared beakers. Each of the beaker was stirred for 1800 s at 500 rpm using Digital magnetic stirrer (UTech, India) to get the homogenous solution. The aforementioned steps were repeated and two more sets (each of 06 beakers) were prepared as a result a total of 18 sample of homogenous solution were prepared. Each set (06 samples) was heated in the domestic LG microwave oven (MH2046HB, India) for different duration 165 s, 225 s and 300 s. The nomenclature of these samples are given in Table 1.

After heating, each of the beaker is taken out and 20 ml distilled water is poured into it to liquidate the obtained matter and obtained solution is collected in another beaker by filtration through whatman filter paper (2–5 μm). The obtained filtered solution was then sonicated for 1800 s using Citizen ultrasonic cleaner (DSA, India) followed by the centrifugation at 10,000 rpm for 1800 s using REMI centrifuge (MM-12 C, India) so that heavier particles get settle down. Finally, supernatant of this solution is filtered through a syringe polytetrafluoroethylene (PTFE) membrane (0.22 μm). This is referred to as purified stock EDTA-CQDs solution and is used for different characterizations with necessary dilutions wherever required. Synthesis process is graphically presented in Figure S1. In case where powder EDTA-CQDs are required, the PTFE filtered supernatant is kept in the hot air oven at 40 °C for 12 h to ensure the gradual evaporation of water molecules. The resultant matter was dried and ground in mortar pastel to get the fine powder of EDTA-CQDs.

**Table 1:** Nomenclature of samples before and after heating.

S. no.	Samples (Pre-heat) [CA + Urea + EDTA]	EDTA (g)	Synthesis duration (s)	Samples (Post-heat) CQDs	
1	Sample 1	0.0	165	E1-CQD@165sec	EDTA-CQD@165sec
2	Sample 2	0.2	165	E2-CQD@165sec	
3	Sample 3	0.4	165	E3-CQD@165sec	
4	Sample 4	0.6	165	E4-CQD@165sec	
5	Sample 5	0.8	165	E5-CQD@165sec	
6	Sample 6	1.0	165	E6-CQD@165sec	
7	Sample 7	0.0	225	E1-CQD@225sec	EDTA-CQD@225sec
8	Sample 8	0.2	225	E2-CQD@225sec	
9	Sample 9	0.4	225	E3-CQD@225sec	
10	Sample 10	0.6	225	E4-CQD@225sec	
11	Sample 11	0.8	225	E5-CQD@225sec	
12	Sample 12	1.0	225	E6-CQD@225sec	
13	Sample 13	0.0	300	E1-CQD@300sec	EDTA-CQD@300sec
14	Sample 14	0.2	300	E2-CQD@300sec	
15	Sample 15	0.4	300	E3-CQD@300sec	
16	Sample 16	0.6	300	E4-CQD@300sec	
17	Sample 17	0.8	300	E5-CQD@300sec	
18	Sample 18	1.0	300	E6-CQD@300sec	

#### UV-Vis absorption spectroscopy

Small amount of purified stock EDTA-CQD solution was appropriately diluted and sonicated for 15 min to ensure the proper distribution of nanomaterials. The concentration of all the samples was kept identical for comparative analysis. The UV-Vis absorption of samples was investigated by double-beam UV-Vis Spectrophotometer (Perkin Elmer-Lambda 35, USA) where the absorption spectrum was recorded from 200 to 700 nm.

#### Thermogravimetric analysis

Thermal stability of EDTA-CQDs was investigated using Thermal Analysis system (Hitachi-STA7300, India). 5-7.5 mg of powder EDTA-CQDs sample was placed in an uncovered crucible and TG signals were recorded over ~ 20 –600 °C at a heating rate of 10 °C/min in the purge gas (N_2_) flow rate of 150 ml/min.

#### Dynamic light scattering (DLS) for particle distribution

Small amount of purified stock EDTA-CQDs solution was appropriately diluted with distilled water and sonicated for 15 min to ensure the proper distribution of nanomaterials. Particle size distribution of the samples was determined using DLS System (Malvern- Zetasizer Nano series UK) with refractive index and absorption set at 1.69 and 1, respectively. Particle Size Distribution intensity (%) is plotted with respect to particle size.

#### Cyclic voltammetry (CV)

A three-electrode configuration in a dimethylformamide (DMF) solution containing the CQDs sample (0.1 mg/ml) and tetrabutylammonium hexafluorophosphate (NBu_4_PF_6_) as the supporting electrolyte (0.1 M). I-V curve was obtained using potentiostat / galvanostat Electrochemical system (Metrohm-Autolab PGSTAT30, Netherlands). A platinum disc (d = 1 mm) embedded in Teflon column, a platinum sheet and a commercial Ag/AgCl electrode being served as the working, counter and the reference electrodes respectively. The solutions were stirred and deaerated by bubbling nitrogen for 2 min prior to each voltammetric measurement and the scan rate was fixed at 0.01 V/s.

#### Fluorescence spectroscopy

Small amount of purified stock EDTA-CQDs solution was appropriately diluted and sonicated for 15 min to ensure the proper distribution of nanomaterials. The concentration of all the samples was kept identical for comparative analysis. PL of the samples was carried by UV-Vis Spectrofluorophotometer (Shimadzu - RF-5301 PC, Japan). The excitation wavelength was varied from 300 to 360 nm and maximum PL was observed at 350 nm. The emission is recorded in the visible spectrum between 400 and 700 nm.

#### Raman spectroscopy

Small amount of purified stock EDTA-CQDs solution was sonicated for 15 min to ensure the proper distribution of nanomaterials and few drop were casted on the silicon substrate and let it for drying at 60–70 °C on hot plate for 30 min. Raman spectrum was measured by Raman spectrometer (Oxford Instrument, Witec confocal microprobe) system with 532 nm Laser.

#### High resolution-transmission electron microscopy (HR-TEM)

Highly-diluted purified EDTA-CQDs droplet was put on the carbon coated (film thickness < 10 nm) grid and dried for 5 min under ambient condition. This TEM sample was inspected by JOEL High Resolution transmission electron microscope (JEM-2100 Plus JOEL, Japan) working at 200 kV.

#### FT-IR spectroscopy

Functional groups of EDTA-CQDs was investigated using FTIR (Bruker Alpha-E, Germany). Small amount of powder EDTA-CQDs sample was used and the signals were recorded over 4000–500 cm^−1^.

#### Cyclic voltammetry (CV)

Small amount of purified stock EDTA-CQDs solution was sonicated for 15 min to ensure the proper distribution of nanomaterials and few drop were casted on the glass substrate and let it for drying at 60 °C in hot air oven for 3 h. XRD measurements were carried out using X-ray diffractometer (Bruker-AXS – D8 Discover, America) having CuKα incident beam having λ = 1.54Å.

#### X-ray photoelectron spectroscopy (XPS)

Elemental analysis was carried out on powder samples using XPS Spectrophotometer (Kratos Analytical Ltd-AXIS Supra, UK) having monochromatic X-ray source (Al Kα).

## Results and discussion

**Fig. 1:**
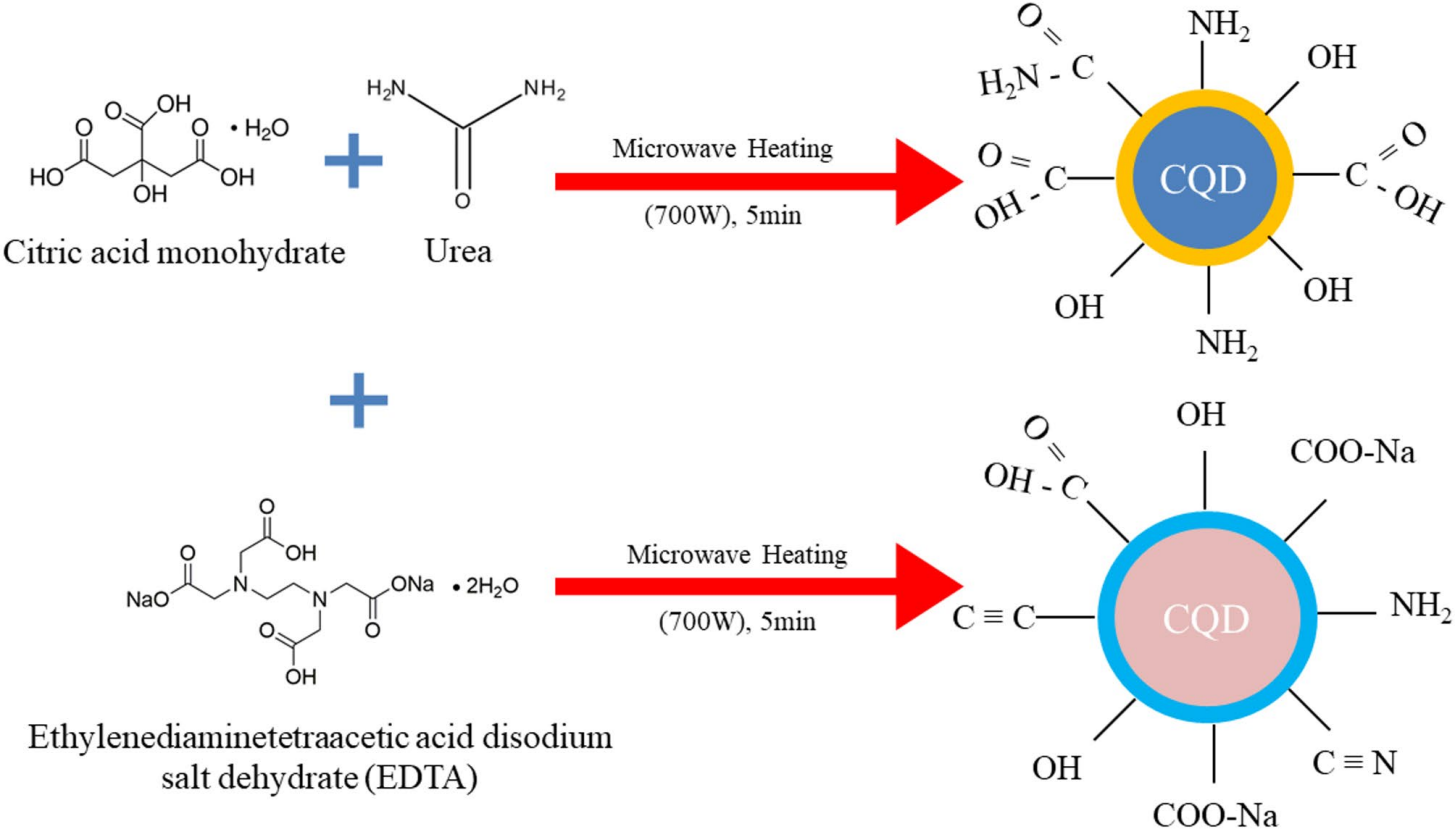
Reaction scheme depicting the changes in CQD size, surface and core after EDTA.

Figure 1 illustrates the schematic representation of carbon quantum dots (CQDs) synthesized via the microwave method, exhibiting a core–shell architecture. The outer shell is functionalized with diverse surface groups, while the inner region consists of a graphitic core. Incorporation of EDTA leads to modification of the surface states through the introduction of sodium-containing moieties. Furthermore, partial penetration of sodium into the core induces structural alterations within the graphitic framework. The localization of sodium on the surface was corroborated by XPS while EDX and XRD provided evidence for sodium incorporation and associated changes within the core structure. The detailed results of these characterizations are elaborated in the subsequent sections of this article.

**Fig. 2:**
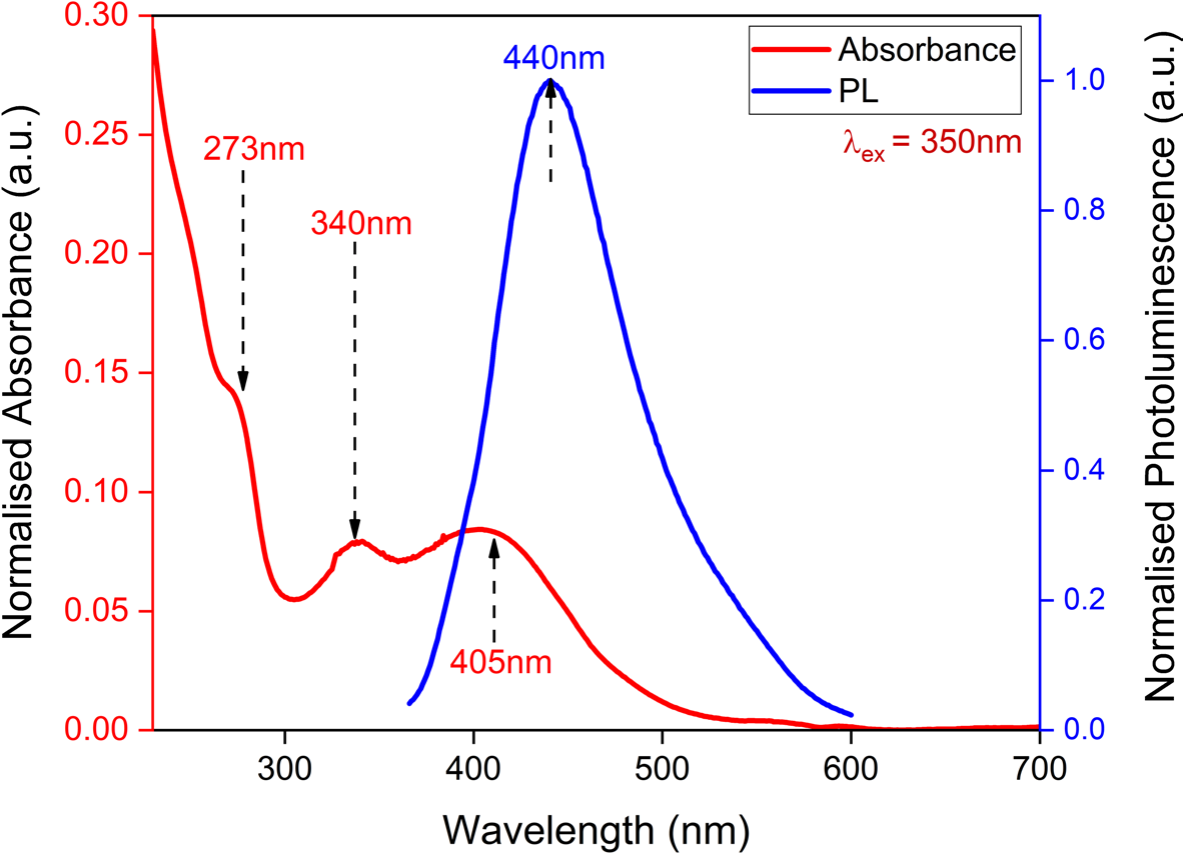
Absorption and PL spectrum of E1-CQD@300sec.

**Fig. 3:**
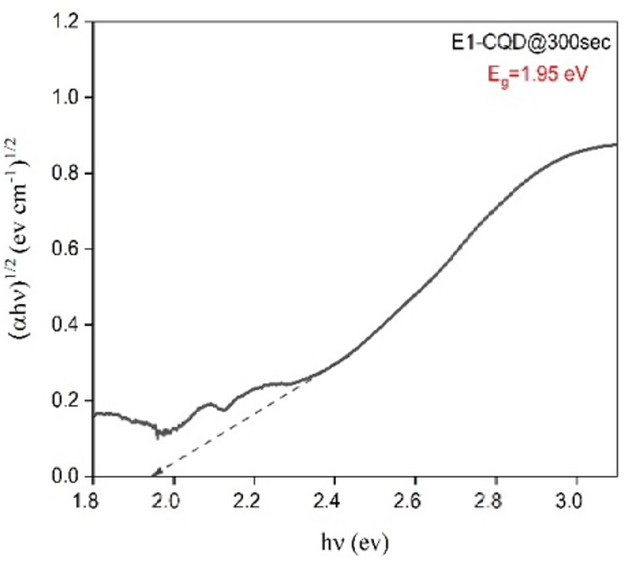
Bandgap from Tauc Plot of E1-CQD@300sec.

In the initial phase the CQDs without EDTA i.e. E1-CQD@300sec were synthesised and its structural properties were studied. The absorption spectrum shown in Fig. 2 reflects the shoulder at ~ 275 nm originating from π– π* transitions of C = C in the Graphitic N^33,34^ whereas the peaks at ~ 340 nm (peak 1) and ~ 405 nm (peak 2) corresponds to the n–π* transition of the C = O at surface and pyridine rings^34,35^. Long tail extended in the visible region of the absorption spectrum resulted from lower energy surface function groups^36^. E1-CQD@300sec exhibited maximum PL intensity at ~ 440 nm upon excitation at 350 nm as seen in Fig. 1. Distant UV-absorption contribute very low PL and majority is contributed by the absorption band near ~ 340 nm associated with the n–π* transition. The bandgap of E1-CQD@300sec was found to be 1.95 eV when evaluated from the tauc plot as shown in Fig. 3. The observed optical characterization was consistent to what was reported earlier by our group^37^.

**Fig. 4:**
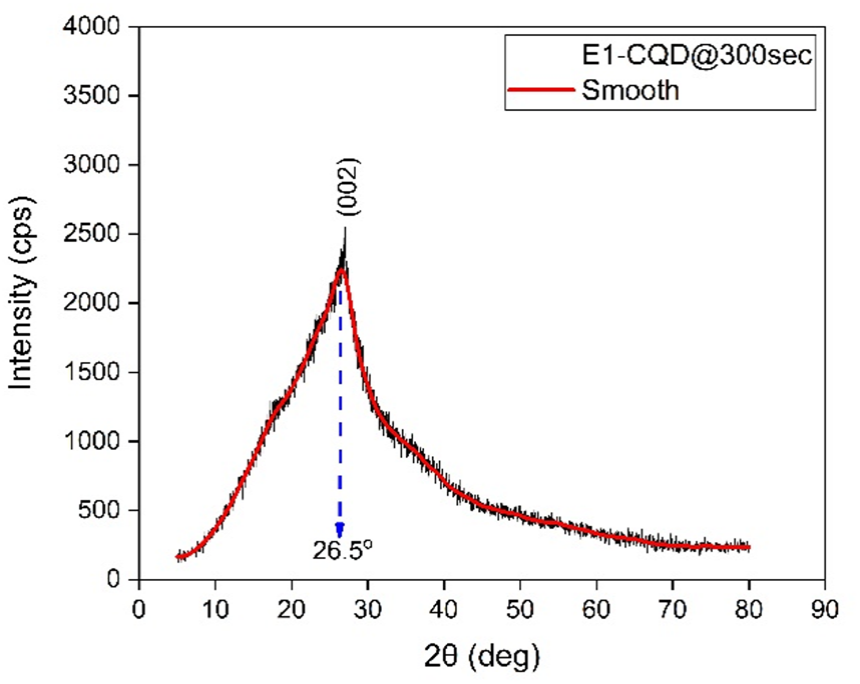
XRD spectrum of E1-CQD@300sec.

**Fig. 5:**
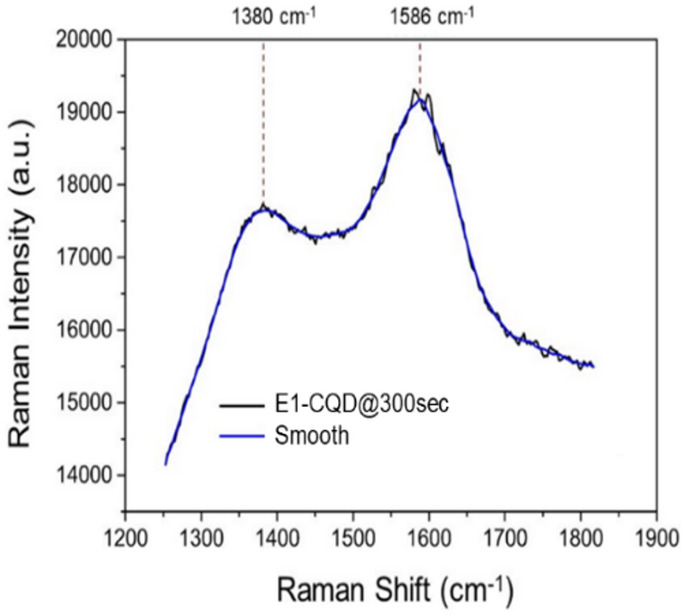
Raman spectrum of E1-CQD@300sec.

To further investigate the structural and chemical features of the E1-CQD@300sec, we carried out XRD and Raman analyses. As shown in Fig. 4, E1-CQD@300sec displays a broad peak around ~ 26.5°, suggesting that these CQDs are amorphous and possess highly disordered structures. This disorder was possibly due to the oxidization of the carbon-carbon bonds in the precursors^38^. In Raman spectra, two characteristic peaks were observed at ~ 1380 and ~ 1586 cm^−1^, representing the D- and G-band respectively (Fig. 5). The D-band is usually referred to as disordered band or defect band which represents the defects in graphitic structure (related to sp^3^ carbon atoms), while the G-band corresponds represents the orderliness in the graphitic structure (related to sp^2^ carbon atoms)^39^. The intensity ratio of the D- and G-bands (I_D_/I_G_) are used to evaluate the degree of disorderliness/structural defect of carbon materials^40^, which was found to be 1.11 (> 1) for this case that indicates high degree of disorderliness which is consistent with the XRD result. It is to be noted that the intrinsic nature of carbon precursors could also affect the structural properties of the CQDs^38^.

**Fig. 6:**
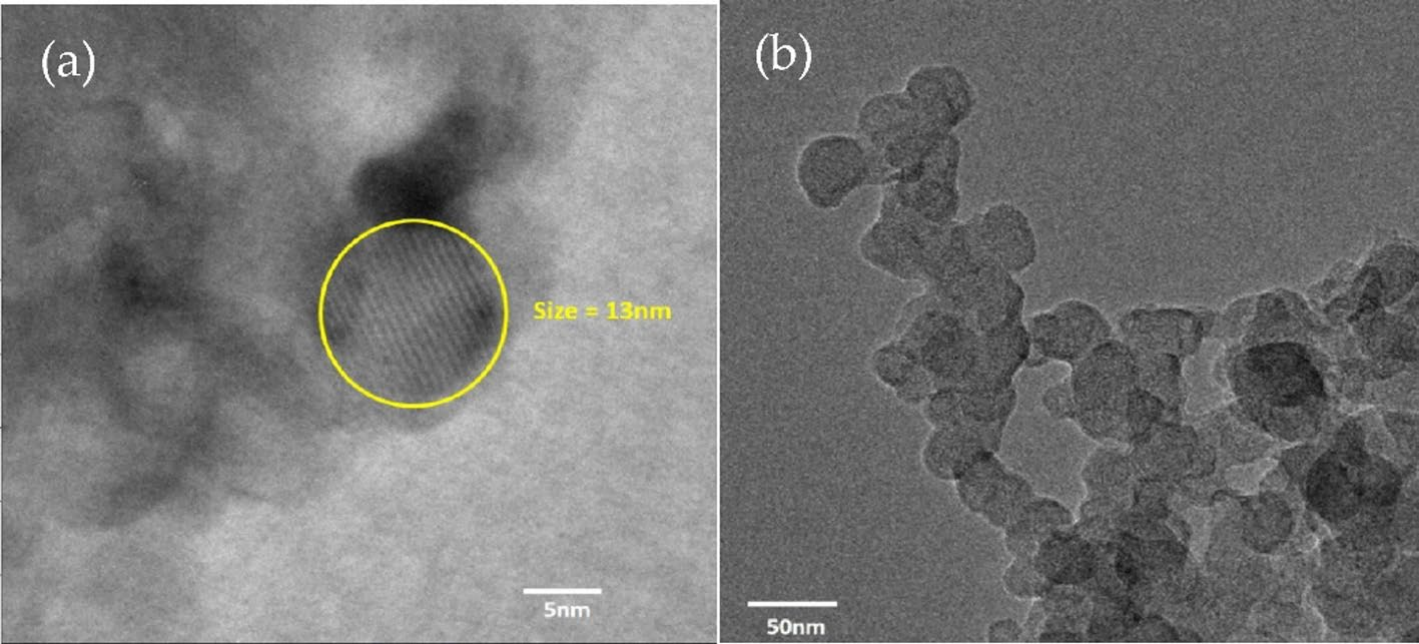
HR-TEM of E1-CQD@300sec  (**a**) single spherical CQD of size ~ 13 nm and (**b**) indicating agglomeration.

The HR-TEM images of E1-CQD@300sec are shown in Fig. 6a, indicating CQDs are spherical in morphology with size ~ 13 nm which is consistent with other reports^41,42^. Due to small size of CQDs they tend to agglomerate which is visible from Fig. 6b. Aggregation may be also caused by drying process during sample preparation for TEM analysis^43^.

In the previous study, the optimized synthesis time was found to be 300 s when EDTA was not present. Whereas, in the present scenario, the EDTA is introduced and optimized synthesis time may change and hence need to be analysed. For this, comprehensive study was carried out where the optical properties were recorded for different concentrations of EDTA at each synthesis duration (165 s, 225 s and 300 s).

The exact position of the peaks of absorption spectrum (peak 1 and peak 2) varies with synthesis duration as per the report^37^ but the variation in small ± 3 nm. Thus, we will be refereeing to peaks instead of its exact value.

**Fig. 7:**
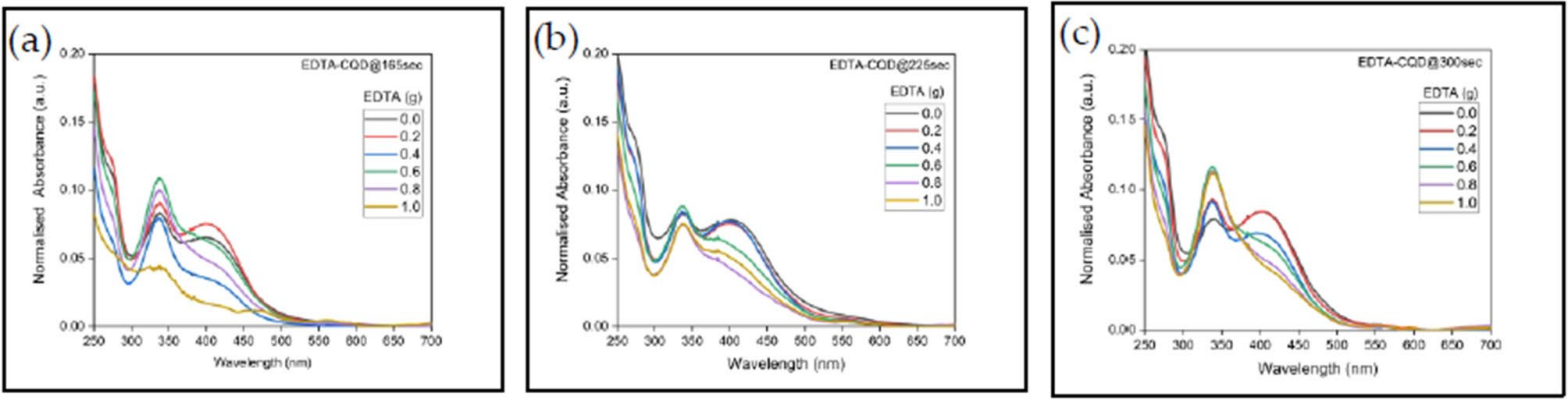
Absorbance of **(a)**. EDTA-CQD@165sec. **(b).** EDTA-CQD@225sec and **(c).** EDTA-CQD@300sec.

The effect of EDTA concentration on the absorbance features of EDTA-CQDs is summarized in Fig. 7a–c. In all cases, peak 1 is associated with C = O groups, while peak 2 corresponds to pyridine-related nitrogen states.

In EDTA-CQD@165sec, the intensity of peak 1 increases with the EDTA concentration from 0.0 to 0.8 g, likely due to the enhanced presence of C = O group introduced by the EDTA (Fig. 7a). However, at 1.0 g, the intensity drops sharply, and the underlying cause of this decrease remains unclear. In contrast, intensity of peak 2 decreases with increasing EDTA concentration (0.0–1.0 g) except for EDTA 0.2 g Peak 2 disappears completely at concentrations ≥ 0.8 g, most likely due to the replacement of nitrogen atoms with sodium, leading to a reduction in pyridine groups. Overall, net absorbance decreases with increasing EDTA concentration (≥ 0.2 g), with the maximum value observed at 0.2 g. Consequently, E2-CQD@165sec exhibits the highest absorbance among the EDTA-CQD@165sec samples (Table S1).

For EDTA-CQD@225sec, peak 1 increases steadily up to 0.6 g, while peak 2 diminishes continuously and vanishes at 1.0 g, consistent with the reduction in pyridine groups caused by sodium substitution. The overall absorbance decreases with concentration, with the maximum at 0.0 g (E1-CQD@225sec).

For EDTA-CQD@300sec, peak 1 increases up to 0.6 g and then stabilizes, in line with EDX results showing a plateau in oxygen content beyond this concentration. Peak 2 decreases with concentration and disappears at ≥ 0.6 g, again reflecting a loss of nitrogen content. The maximum absorbance is recorded at 0.0 g (E1-CQD@300sec).

In summary, increasing EDTA concentration generally enhances C=O-related peak 1 (up to 0.6–0.8 g) while suppressing nitrogen-related peak 2, which disappears at higher concentrations. The net absorbance decreases with EDTA concentration in all samples, with the maximum values observed at 0.2 g for 165 s and at 0.0 g for both 225 s and 300 s (Table S1).

On detailed comparison it was found that E1-CQD@300sec exhibit the highest absorbance amongst EDTA-CQDs and absorbance of EDTA-CQD@300sec > EDTA-CQD@225sec > EDTA-CQD@165sec in most of the EDTA concentrations as seen from Table S1. It may be concluded that introduction of EDTA certainly affects the absorbance of synthesized EDTA-CQDs but it is the heat duration that finally decides the density of states of CQDs. The increase in absorbance with heat duration is in consistency with earlier report^37^.

**Fig. 8:**
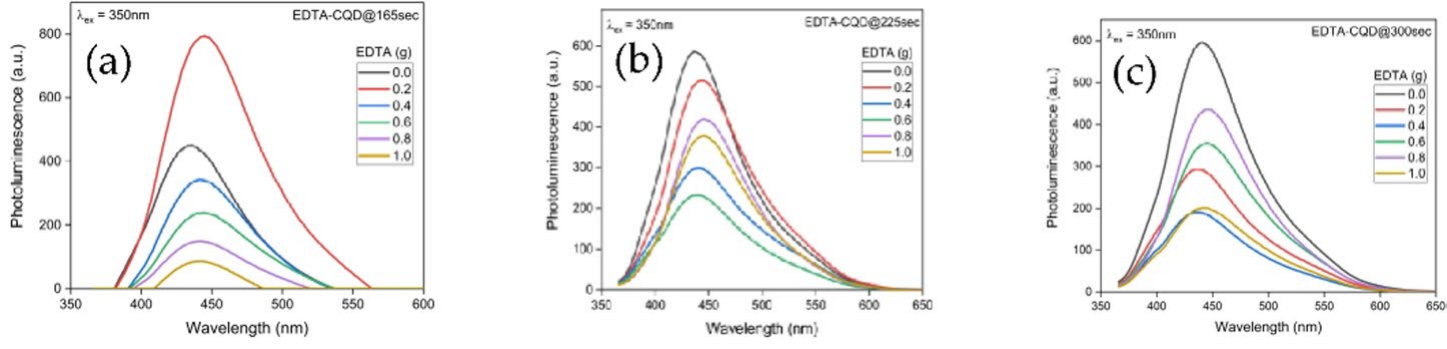
PL of **(a)** EDTA-CQD@165sec, (b) EDTA-CQD@225sec and (c) EDTA-CQD@300sec.

The photoluminescence (PL) spectra of EDTA-CQD@165sec, EDTA-CQD@225sec, and EDTA-CQD@300sec were recorded at an excitation wavelength (λ_ex_) of 350 nm. As shown in Fig. 8a–c, the PL intensity is influenced by EDTA concentration, generally decreasing with the increasing concentration though some deviations from this trend are evident (Figure S2). This is complementary to what have been reported by Zhenxu Lin., et al. where they observed the enhanced PL with Na doping^26^. A slight shift in the PL emission wavelength (± 10 nm) was also observed with varying EDTA concentrations.

The maximum PL emission was recorded at ~ 444 nm for E2-CQD@165sec, ~ 438 nm for E1-CQD@225sec, and ~ 440 nm for E1-CQD@300sec. The far-UV absorption peak contributes minimally to the PL, with the visible-region emission arising primarily from absorption near 340 nm^25^. This excitation-dependent behavior can be attributed to the Franck–Condon principle, which describes how absorption and fluorescence depend on the overlap between vibrational states of the ground and excited electronic levels.

Overall, the PL intensity followed the order: E2-CQD@165 s > E1-CQD@225 s ≈ E1-CQD@300 s (Figure S2). While the relative intensity of EDTA-CQD@300 s is not consistently higher than that of the other samples, its PL behavior remains relatively stable and comparable across different EDTA concentrations.

As observed, the absorbance and PL was highest for E1-CQD@300sec and E2-CQD@165sec respectively. There is a trade-off between the selection for the highest absorbance or highest PL CQDs.

The optical analysis revealed that the highest absorbance was obtained for E1-CQD@300sec, while the strongest PL emission was observed for E2-CQD@165sec, indicating a trade-off between maximizing absorbance and PL intensity. Notably, EDTA-CQD@300sec exhibited consistently moderate-to-high PL across all EDTA concentrations, along with the highest absorbance, making it the most suitable candidate from a photovoltaic perspective. Since CQDs with strong light-harvesting ability and appreciable PL are expected to enhance solar cell performance, EDTA-CQD@300sec was identified as possessing the most favorable balance of optical properties. This synthesis time (300 s) is consistent with previous reports^37^, suggesting that the synthesis duration exerts a more critical influence than EDTA concentration on the optical behavior of the CQDs. Importantly, the combination of high absorbance and stable PL intensity makes EDTA-CQD@300sec particularly promising for integration into organic solar cells. Accordingly, further detailed characterizations—including cyclic voltammetry (CV), dynamic light scattering (DLS), X-ray diffraction (XRD), Fourier-transform infrared spectroscopy (FTIR), and thermogravimetric analysis (TGA)—were carried out on EDTA-CQD@300sec. Representative photographs of the samples under ambient and UV illumination are shown in Figure S2a–c.

**Fig. 9:**
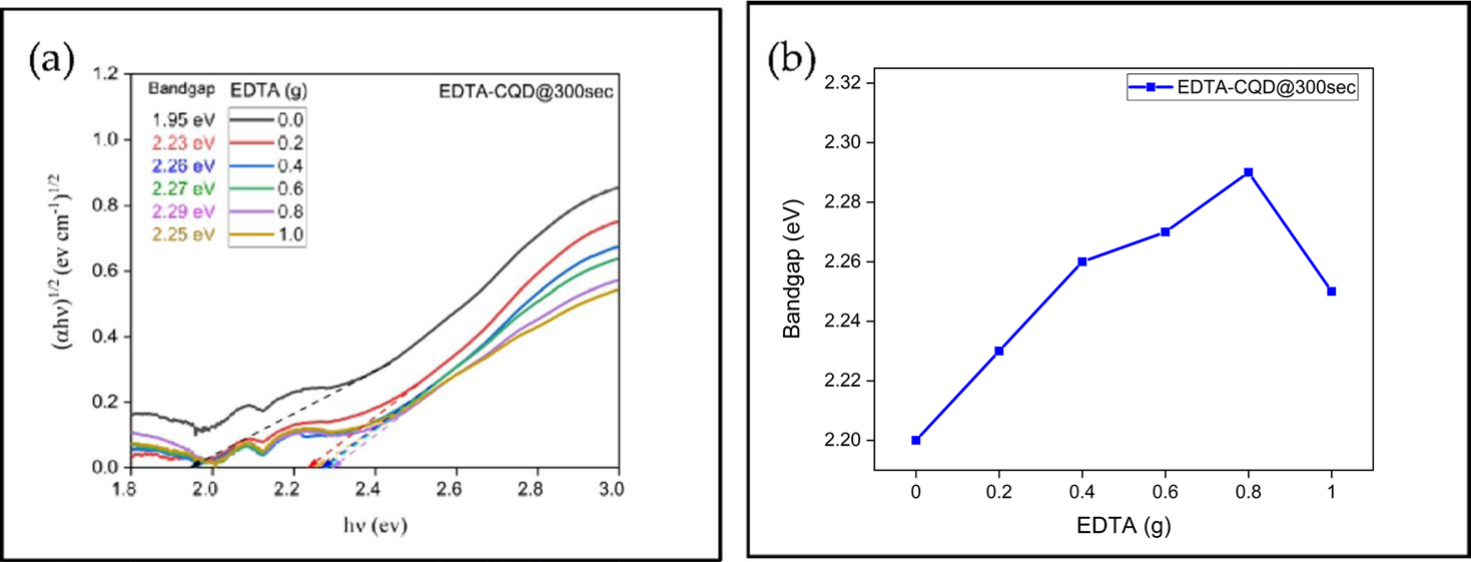
**(a)** Tauc plot of all EDTA-CQD@300sec for deriving their bandgap **(b)** bandgap variation with EDTA.

The bandgap of all the EDTA-CQD@300sec were evaluated from tauc plot as shown in the Fig. 9a. and it was found that bandgap equals to 1.95ev for 0.0 g EDTA, which enhanced with increase in EDTA concentration and reduced finally at 1.0 g as shown in Fig. 9b.

**Fig. 10:**
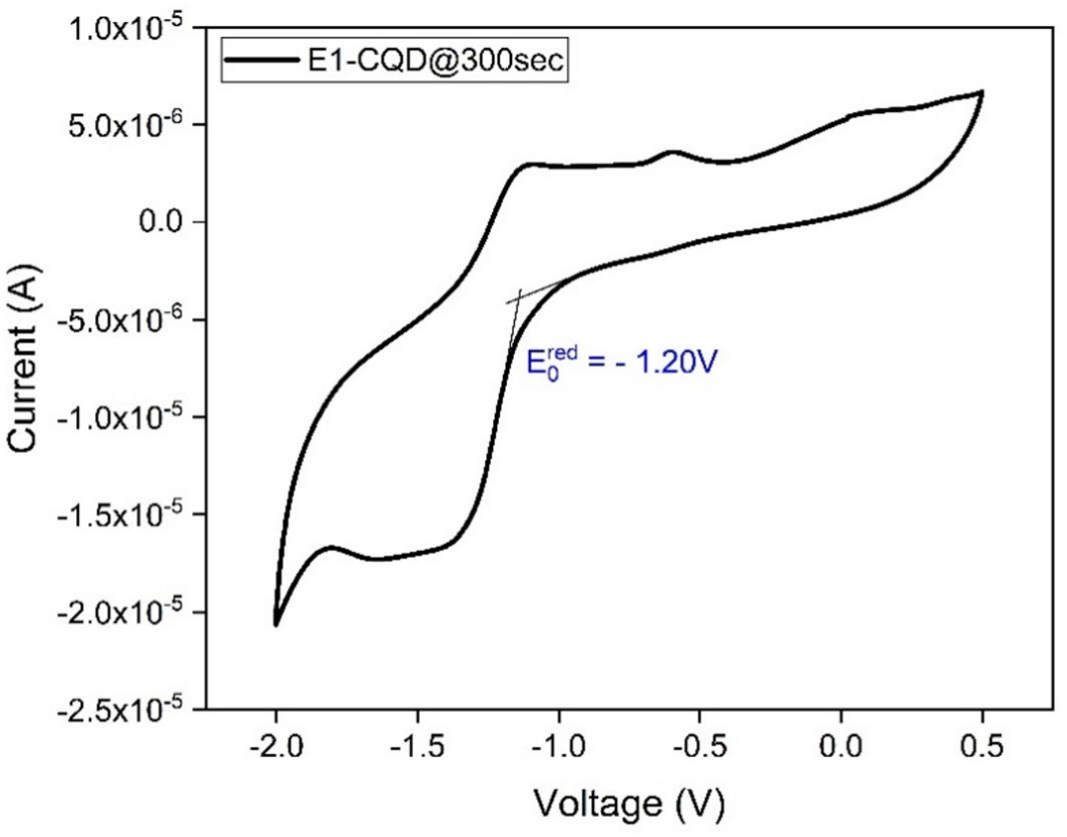
CV plot of E1-CQD@300sec.

The evaluation of highest occupied molecular orbit (HOMO) and lowest occupied molecular orbit (LUMO) energy levels becomes an extremely critical parameter for photovoltaic application. For this purpose, electrochemical CV was carried out for EDTA-CQD@300sec and CV plots were taken. The CV plot of E1- CQD@300sec is shown in Fig. 10 from which it is evident that the onset of reduction potential ($\:{E}_{O}^{red})=-1.20V$. All the remaining CV plots are shown in Figure S3 (a-e) and $\:{E}_{O}^{red}$ of all EDTA-CQD@300sec was calculated likewise.

**Fig. 11:**
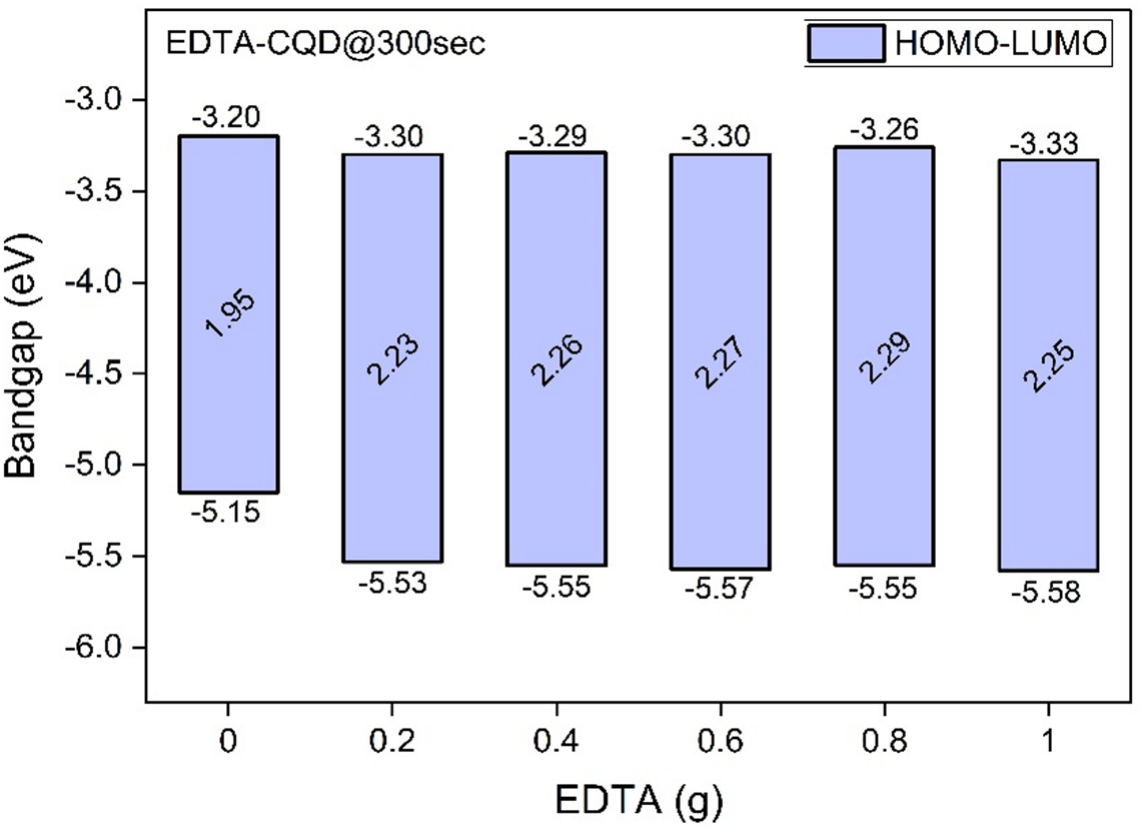
HOMO-LUMO energy levels of EDTA-CQD@300sec.

The obtained onset reduction potential was in compliance with other reports^44,45^. The LUMO and HUMO of all EDTA-CQD@300sec were thus calculated from the information of $\:{E}_{O}^{red}$ and $\:{E}_{g}$ (Optical bandgap) using 4.1 and 4.3^44,45^ and the results are given in the Table 2. The HOMO and LUMO of EDTA-CQD@300sec are shown in Fig. 11.

1 $$\:{\text{E}}_{\text{L}\text{U}\text{M}\text{O}}={-\:(4.4+\text{E}}_{\text{r}\text{e}\text{d}})\text{e}\text{V}$$

2 $$\:{\text{E}}_{\text{o}\text{p}\text{t}\text{i}\text{c}\text{a}\text{l}}={{(\text{E}}_{\text{L}\text{U}\text{M}\text{O}}-\text{E}}_{\text{H}\text{O}\text{M}\text{O}})\text{e}\text{V}$$

3 $$\:{\text{E}}_{\text{H}\text{O}\text{M}\text{O}}={(\text{E}}_{\text{L}\text{U}\text{M}\text{O}}-{\text{E}}_{\text{o}\text{p}\text{t}\text{i}\text{c}\text{a}\text{l}})\text{e}\text{V}$$

**Table 2:** Evaluated HOMO-LUMO energy levels.

CQD Type		EDTA (gm)	$\:{E}_{g}$ (eV)	$\:{E}_{O}^{red}$ (V)	LUMO (eV)	HOMO (eV)
EDTA-CQD@300sec	E1-CQD@300sec	0.0	1.95	-1.20	-3.20	-5.15
	E2-CQD@300sec	0.2	2.23	-1.10	-3.30	-5.53
	E3-CQD@300sec	0.4	2.26	-1.11	-3.29	-5.55
	E4-CQD@300sec	0.6	2.27	-1.10	-3.30	-5.57
	E5-CQD@300sec	0.8	2.29	-1.14	-3.26	-5.55
	E6-CQD@300sec	1.0	2.25	-1.07	-3.33	-5.58

The results shown in the Table 2 indicates significant variation (~ 0.1 eV) in LUMO energy levels (from − 5.15 eV to -5.53 eV) as soon as EDTA (0.2 g) was introduced (E2-CQD@300sec). But, after the incorporation of EDTA, the variation in LUMO energy level with EDTA variation was very small (max. ~0.04 eV) amongst the E2-EDTA@300sec to E6-EDTA@CQD300sec.

Since EDTA contains carbon (C), nitrogen (N), sodium (Na), and oxygen (O) atoms, elemental analysis plays a crucial role in understanding the influence of each element on the properties of EDTA-CQD@300sec. During the energy-dispersive X-ray (EDX) measurements, different regions were examined, and the corresponding peaks for selected samples are shown in Figure S4 (a-d). The atomic and weight percentages of the elements reveal that the carbon content remains relatively constant, while nitrogen decreases and sodium increases with higher EDTA concentrations. Additionally, the atomic and weight percentages of oxygen increase as the EDTA concentration rises.

**Fig. 12:**
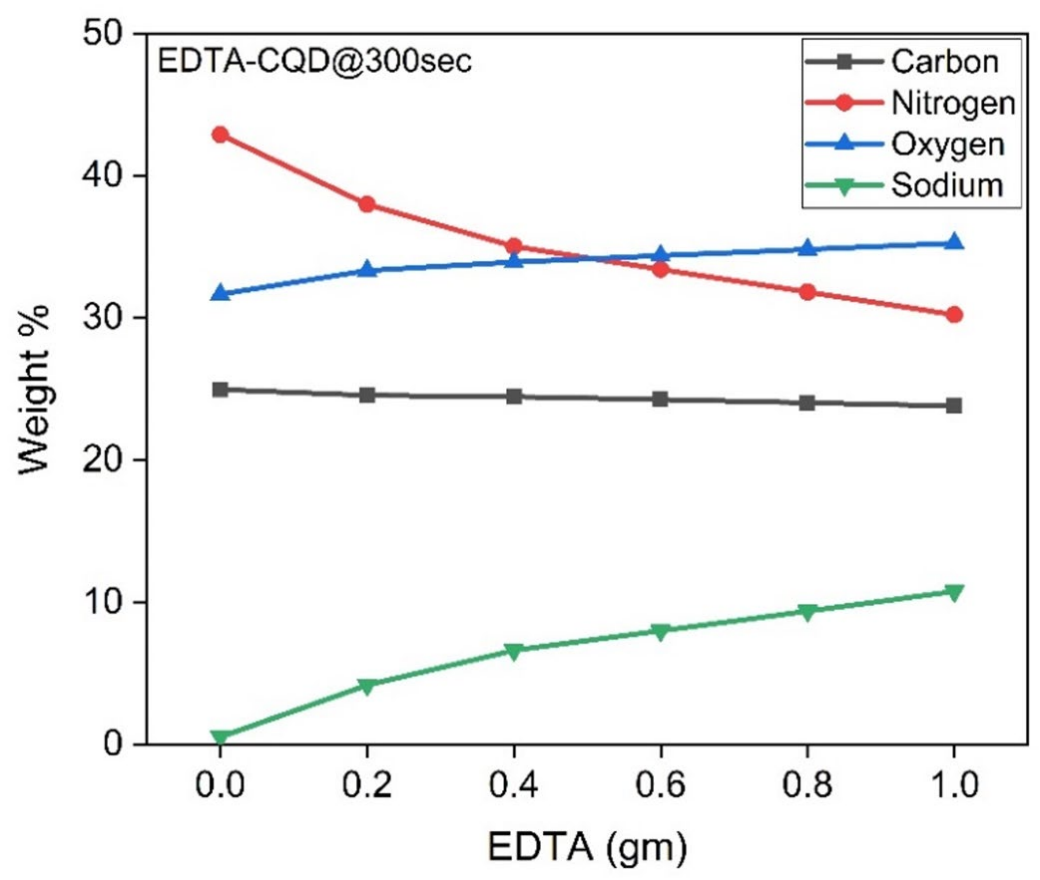
Weight concentration of different elements in EDTA-CQD@300sec with varying EDTA concentration.

The elemental composition (C, N, Na, and O) for all EDTA-CQD@300sec samples is summarized in Table 3, and the trends in these values are illustrated in Fig. 12. Notably, trace amounts of sodium were detected in the E1-CQD@300sec sample, despite the absence of sodium in the precursor materials. This could be attributed to instrumental calibration errors or residual sodium from previous sample analyses. However, it is important to emphasize that no sodium should be present in the E1-CQD@300sec sample, and the reported results reflect the measurements obtained.

**Table 3:** Atomic and weight% of carbon, nitrogen, oxygen and sodium of EDTA-CQD@300sec.

CQD	EDTA	Carbon		Nitrogen		Oxygen		Sodium	
	(gm)	Atomic %	Weight%	Atomic %	Weight%	Atomic %	Weight%	Atomic %	Weight%
EDTA-CQD@300sec	0.0	29.08	24.94	42.88	42.88	27.71	31.65	0.32	0.53
	0.2	29.11	24.54	38.63	37.97	29.67	33.32	2.59	4.17
	0.4	29.31	24.44	36	35.01	30.54	33.92	4.15	6.62
	0.6	29.24	24.23	34.6	33.4	31.14	34.36	5.05	8
	0.8	29.18	24.01	33.13	31.8	31.74	34.8	5.96	9.38
	1.0	29.12	23.79	31.66	30.2	32.34	35.24	6.87	10.76

**Fig. 13:**
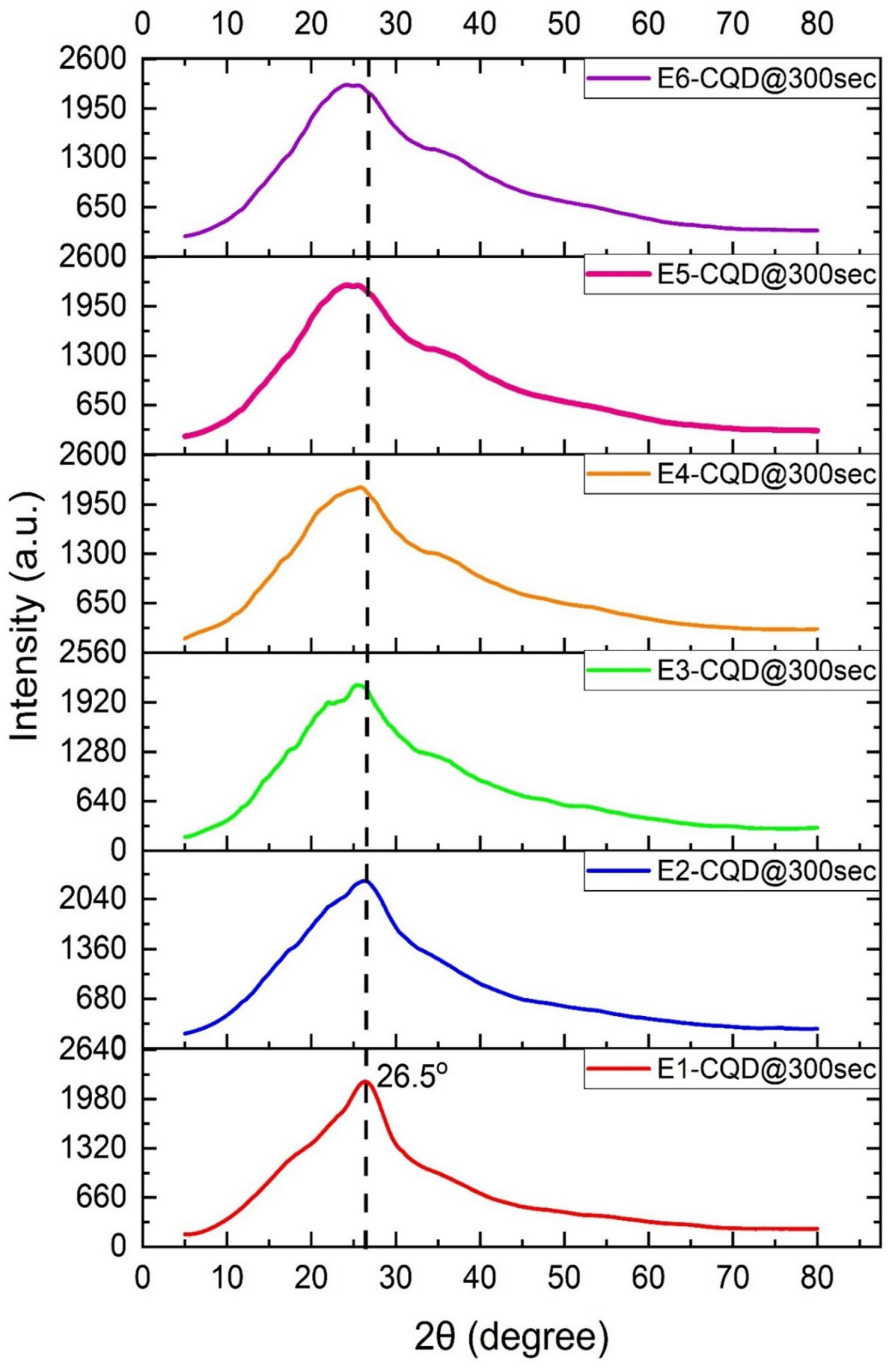
X-ray diffraction spectrum of EDTA-CQD@300sec.

XRD analysis of EDTA-CQD@300sec revealed a broad peak at ~ 26.5° (d = 0.34 nm), corresponding to the (002) plane of low-graphitic carbon (Fig. 13). The pronounced broadening indicates a highly disordered, amorphous structure, further supported by HR-TEM observations. This disorder is attributed to oxidation-induced disruption of carbon–carbon bonds in the precursor, in agreement with earlier studies^38,46,47^.

**Fig. 14:**
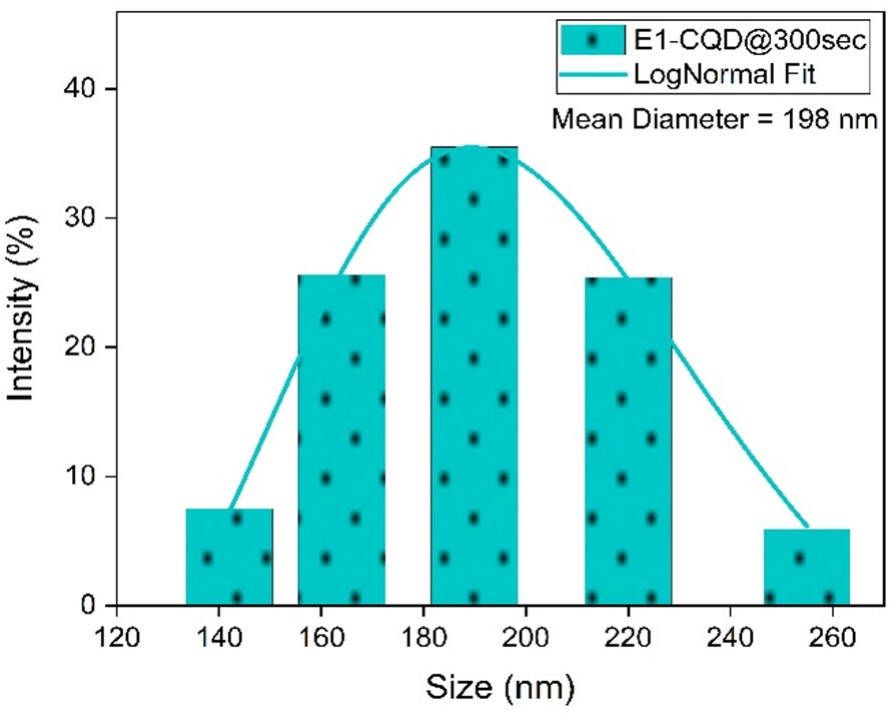
Size distribution of E1-CQD@300sec.

With increasing EDTA concentration, the XRD peak broadens and shifts to lower angles, indicating enhanced structural disorder and increased inter-planar spacing (Fig. 13; Table 4). This broadening is consistent with FTIR and EDX evidence of oxidation (Figs. 12 and 14) and the reduction in crystallite size (Table S2). The expansion in inter-planar spacing may further be attributed to sodium substitution for nitrogen, as suggested by EDX analysis (Table 3).

The crystallite size of EDTA-CQD@300sec was calculated using the Scherrer equation^48^, and the corresponding data are provided in Table S2.

4 $$\:D=\frac{K{\uplambda\:}}{{\upbeta\:}\text{c}\text{o}\text{s}{\uptheta\:}}$$

Where, K is the Scherrer constant or particles shape factor, λ is the wavelength of CuKα radiations, β is FWHM of the maximum intensity peak and θ is the angle obtained from 2θ value corresponding to maximum intensity peak.

**Table 4:** Variation of inter-planar spacing with EDTA concentration.

Sample		EDTA (g)	2θ (degree)	Sin(θ) (radians)	Lattice spacing (d) (nm)
EDTA-CQD@300sec	E1-CQD@300sec	0.0	26.451	0.229	0.337
	E2-CQD@300sec	0.2	26.141	0.226	0.341
	E3-CQD@300sec	0.4	25.541	0.221	0.349
	E4-CQD@300sec	0.6	25.100	0.217	0.355
	E5-CQD@300sec	0.8	24.972	0.216	0.356
	E6-CQD@300sec	1.0	24.851	0.215	0.358

In order to further explore the significant influence of EDTA on the morphology of CQDs, size distribution was calculated via the dynamic light scattering (DLS), which provided a mean hydrodynamic diameter of EDTA-CQD@300sec. The mean diameter of E1-CQD@300sec obtained from DLS data was ~ 198 nm as shown in Fig. 14. This was quite high in contrast to the diameter of ~ 13 nm seen from HR-TEM. This should be the size of agglomerated E1-CQD@300sec because nanoparticles have the tendency to agglomerate due to van der waal interactions and this was observed in HR-TEM results (Fig. 5b) and the same was reported by Reyna et al.^34^. The size was further increased in the presence of EDTA, with mean diameter of 245 ± 2 nm and highest mean value of 399 ± 4 nm for E2-CQD@300sec and E5-CQD@300sec respectively. The increase in size in the presence of EDTA may be due to following reasons: (1) uncontrolled nucleation caused by EDTA, (2) EDTA-CQD complex formation, (3) high concentration of EDTA, (4) insufficient surface coverage by EDTA.

The particle size of EDTA-CQD@300sec increased with EDTA concentration, reaching a maximum at 0.8 g (E5-CQD@300sec) before decreasing to ~ 299 nm at 1.0 g (E6-CQD@300sec). The size distribution of E2-CQD@300sec to E6-CQD@300sec is shown in the Figure S5(a-e). This reduction at higher EDTA levels is likely due to the breakup of larger aggregates into smaller, more stabilized EDTA–CQDs. Thus, the agglomerate size is primarily influenced by van der Waals interactions, EDTA concentration, and available binding sites on the CQDs. Mean diameter of EDTA-CQD@300sec at different EDTA values is shown in the Figure S6.

**Fig. 15:**
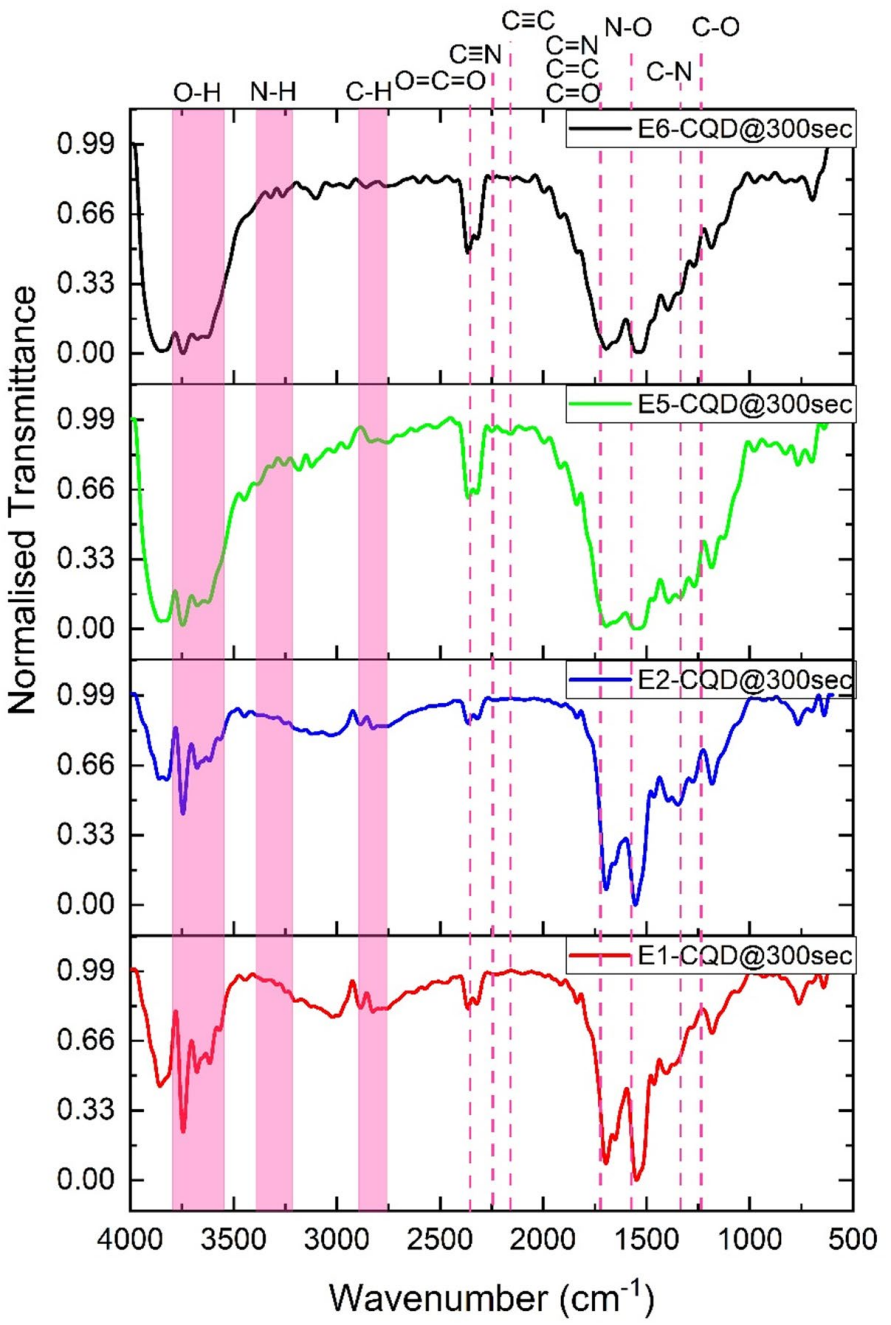
FTIR spectra of EDTA-CQD@300sec for 0.0 g, 0.2 g, 0.8 g and 1.0 g EDTA.

FT-IR spectra of EDTA-CQD@300sec for EDTA equals 0.0, 0.2, 0.8, and 1.0 g shown in Fig. 15. Peaks at 1183 and 1269 cm^−1^ correspond to C–O and C–N stretching, while the band at 1150 cm^−1^ is attributed to N–O stretching. The vibrations at 1694 cm^−1^ arise from C = O and C = C bonds^49–51^, and bands at 2155 and 2244 cm^−1^ indicate C ≡ C and C ≡ N groups. Peaks in the 2775–2940 cm^−1^ region are associated with C–H stretching^52^, whereas bands at 3260–3450 cm^−1^ and 3300–3600 cm^−1^ correspond to N–H and –OH groups, respectively, consistent with previous reports^36,52^. Notably, O = C = O and –OH vibrations become prominent at EDTA above 0.6 g, while C ≡ C and C ≡ N groups are absent without EDTA. Peaks at 1550 and 1694 cm^−1^, assigned to C = C and C = N stretching of pyridine rings, decrease upon EDTA addition, with C = N overlapping and broadening with C = O at 1694 cm^−1^. A broad –OH band around 3800 cm^−1^ emerges with EDTA, accompanied by a decrease in C = N and increased C = O intensity. The observed broadening of the FTIR band in the 1400–1700 cm^−1^ region with increasing EDTA concentration arises from enhanced COO⁻–Na⁺ interactions, reflecting greater sodium incorporation into the CQDs.

**Fig. 16:**
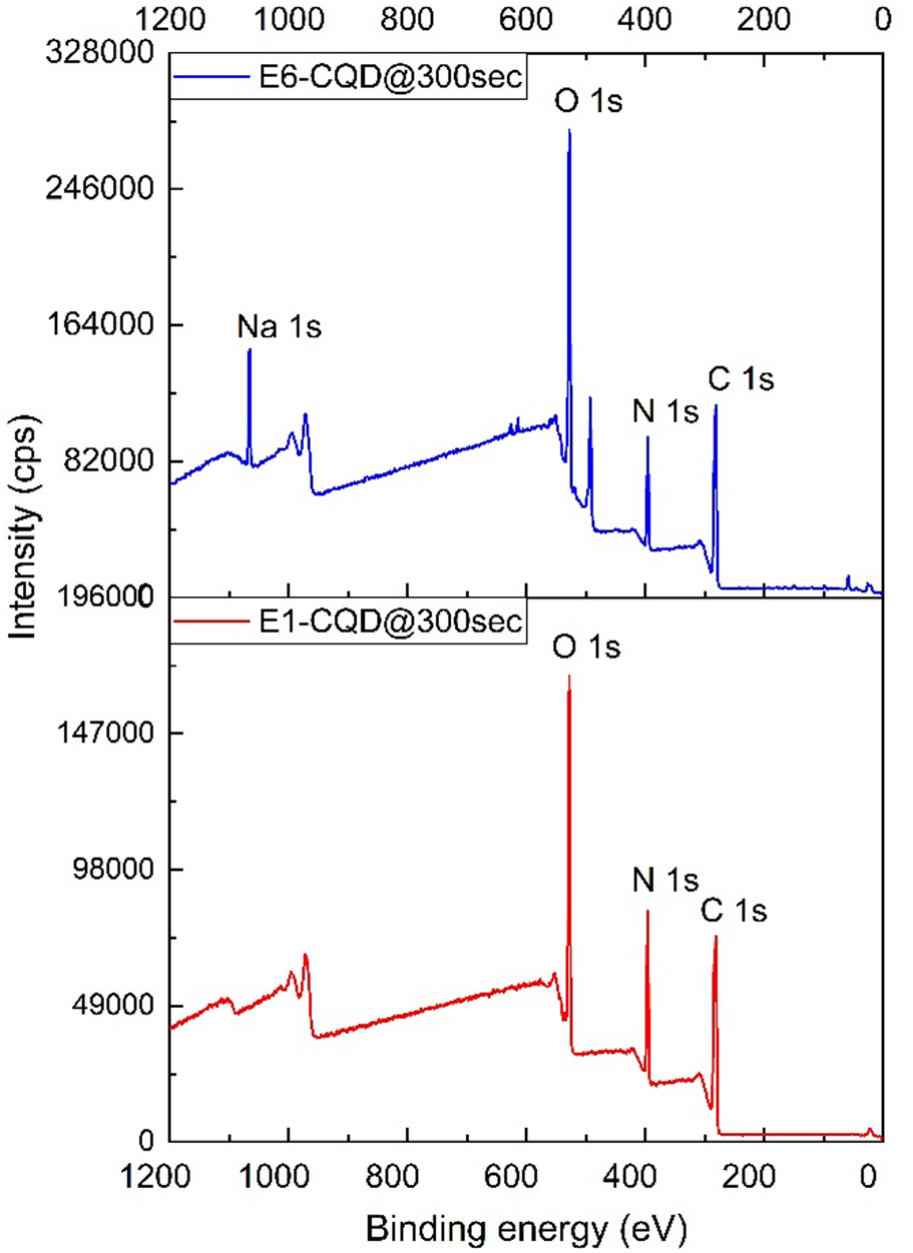
XPS spectra of EDTA-CQD@300sec for 0.0 g and 1.0 g EDTA.

The presence of Na in E6-CQD@300 s was confirmed by XPS analysis (Fig. 16), consistent with EDX results (Fig. 12). With increasing EDTA, the surface oxygen concentration increases, whereas nitrogen decreases, a trend also observed in EDX (Fig. 12). The differences in atomic concentrations between XPS and EDX arise because XPS probes only the surface, while EDX provides bulk-averaged values. The measured atomic and mass concentrations from XPS are summarized in Table 5.

**Table 5:** Summary of elemental concentration of E1-CQD@300sec and E6-CQD@300sec from XPS.

Element	BE [eV]	Atomic conc. [%]	Error [%]	Mass conc. [%]	Error [%]
E1-CQD@300sec					
C 1s	284.4	61.966	0.234	56.368	0.254
O 1s	531.4	21.475	0.172	26.022	0.201
N 1s	399.4	16.494	0.190	17.497	0.201
Na 1s	1071.4	0.065	0.106	0.113	0.185
E6-CQD@300sec					
C 1s	285	56.766	0.207	49.582	0.213
O 1s	531	27.158	0.177	31.598	0.196
N 1s	399	12.333	0.152	12.563	0.155
Na 1s	1069	3.743	0.094	6.258	0.153

The thermal stability of EDTA-CQD@300sec is critical for its potential application in photovoltaic devices. To assess the thermal stability of the synthesized EDTA-CQD@300sec, thermogravimetric analysis (TGA) was performed. The weight loss observed in the TGA data can be classified into two main phases. Phase 1 is an endothermic process, which corresponds to the evaporation and desorption of volatile compounds, while Phase 2 consists of various exothermic processes related to the thermal degradation of the material’s components^53^. The temperature ranges for these phases vary depending on the EDTA concentration.

**Fig. 17:**
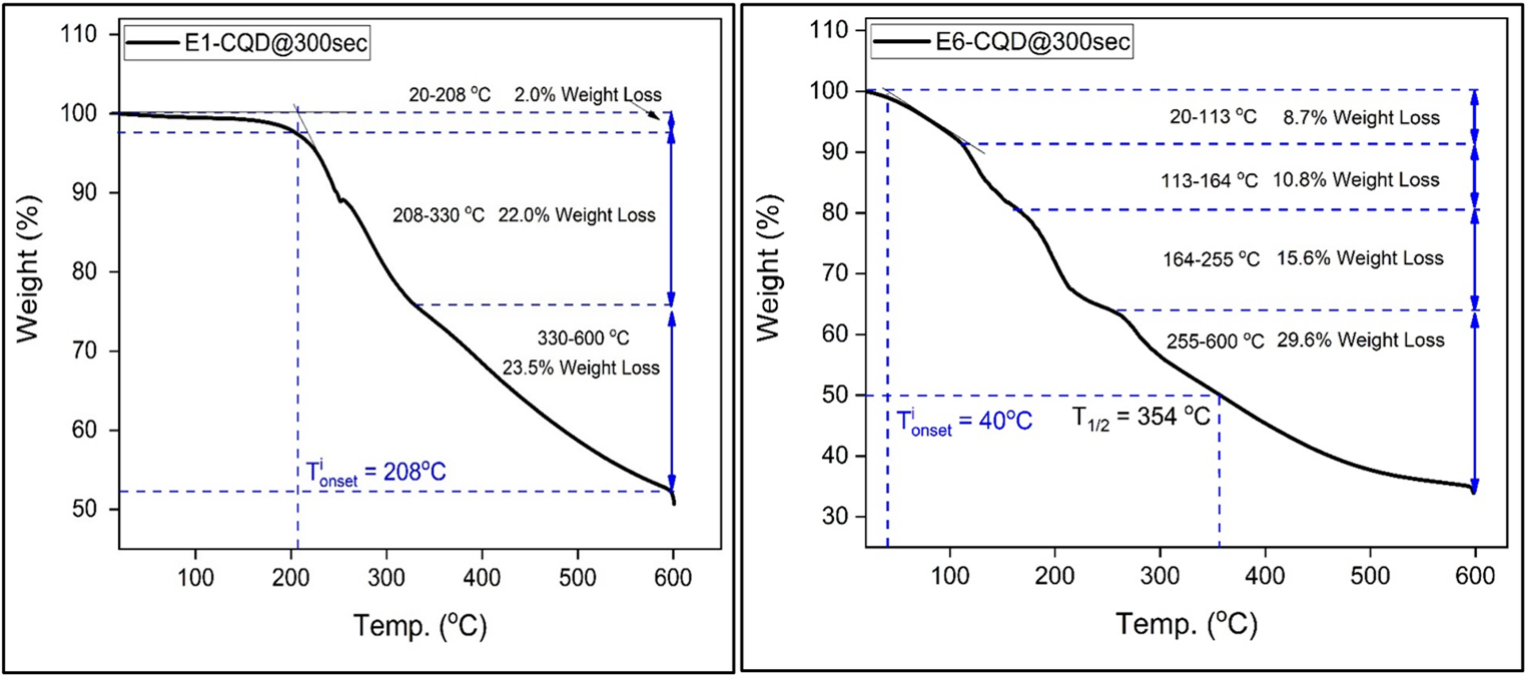
Thermal stability analysis of (**a**) E1-CQD@300sec and (**b**) E6-CQD@300sec.

The TGA results for E1-CQD@300sec (Fig. 17) shows an initial weight loss of a 2% at 208 °C, attributed to the evaporation of adsorbed water molecules, water produced during dehydration reactions, or other volatile compounds^54^ or release of ammonia gas from urea decomposition^55^. The onset temperature of weight loss $\:({T}_{onset}^{i}$), determined using the tangent method, provides an indication of thermal stability.

Between 208 and 330 °C, a 22% weight loss occurs, likely due to decomposition of oxygen-containing functional groups such as C = O, –COOH, and CONH₂^56,57^. A further 23.5% loss between 330 and 600 °C is attributed to degradation of the nitrogen-doped carbon lattice, where heteroatom-induced defects accelerate decomposition relative to pure carbon^58,59^. Extrapolation beyond 600 °C suggests a total weight loss of ~ 50% at ~ 638 °C.

The introduction of EDTA significantly influenced the thermal behaviour of EDTA-CQDs. E2-CQD@300sec showed an initial weight loss of 6.7% at 172 °C (Figure S7a), attributed to faster evaporation and desorption of volatile compounds from EDTA-functionalized CQDs. Subsequent weight losses of 8.6% (172–252 °C) and 12.7% (252–320 °C) correspond to decomposition of oxygen- and sodium-containing functional groups introduced by EDTA, occurring at lower temperatures than in non-functionalized CQDs. Further heating (320–600 °C) led to continued degradation, with 50% weight loss at 530 °C, primarily due to CO release and decomposition of the nitrogen- and sodium-doped carbon lattice; sodium incorporation accelerates lattice degradation by weakening the carbon structure^28,58,59^.

**Fig. 18:**
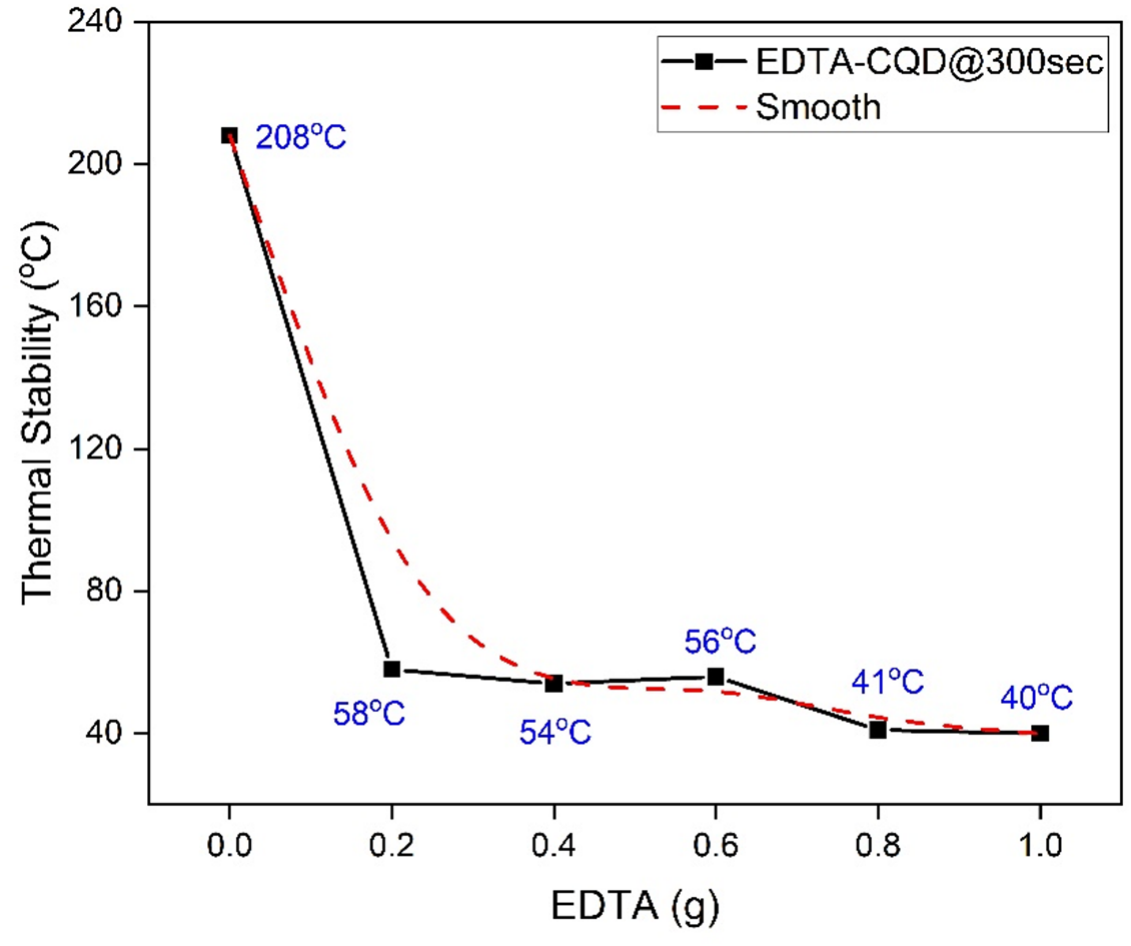
Falling of thermal stability of EDTA-CQD@300sec with EDTA.

With higher EDTA, E3-CQD@300sec exhibited greater initial weight loss (12.2% at 196 °C), followed by continuous losses of 7.6% (196–259 °C), 10.8% (259–312 °C), and 26.4% (312–600 °C) (Figure S7b). Similar trends were observed for E4-CQD@300sec and E5-CQD@300sec as shown in Figure S7c and Figure S7d respectively. Overall, TGA results indicate that increasing oxygen and sodium functionalization via EDTA accelerates thermal degradation, reducing the thermal stability of EDTA-CQDs, consistent with the previous report^28^. The 50% weight loss temperatures for all samples are summarized in Figure S8, with the overall trend shown in Fig. 18.

## Conclusion

Nitrogen- and sodium-doped EDTA-CQDs were successfully synthesized via a one-pot microwave method, yielding CQDs with tunable structural and optical properties. EDTA-CQD@300sec exhibited the highest absorbance and reasonable photoluminescence, making it the most promising candidate for photovoltaic applications. TEM and DLS analyses revealed agglomeration, with particle size influenced by EDTA percentage, van der Waals interactions, and available surface binding sites. XRD confirmed an amorphous, low-graphitic structure, while FTIR, XPS, and EDX analyses demonstrated the incorporation of oxygen and sodium by EDTA, with corresponding modulation of surface functional groups. The bandgap and HOMO–LUMO energy levels could be tuned by varying EDTA concentration, with values ranging from 1.95 to 2.29 eV. Thermal analysis showed that EDTA-CQD@300sec maintained stability up to 208 °C, decreasing with higher EDTA content due to enhanced oxygen and sodium functionalization. Overall, this one-step synthesis combines doping and surface passivation in a green and scalable manner, producing CQDs with a favorable balance of optical and thermal properties suitable for organic photovoltaics and other optoelectronic applications.

## Supplementary Information

Below is the link to the electronic supplementary material.

Supplementary Material 1

## Data Availability

Most of the data obtained and/or analysed is reported in the manuscript and the supplementary file.The raw datasets used and/or analysed during the current study available from the corresponding author on reasonable request.
