# Supplementary material for: Impact of sodium doping on the structural properties of CQDs
Source: Sci Rep. 2025 Nov 19;15:40725. doi: 10.1038/s41598-025-24609-0 (PMC12630768; doi:10.1038/s41598-025-24609-0)
Supplement: Supplementary file 1 — Supplementary Material 1 [file 41598_2025_24609_MOESM1_ESM.docx]

**Supplementary Information**

**
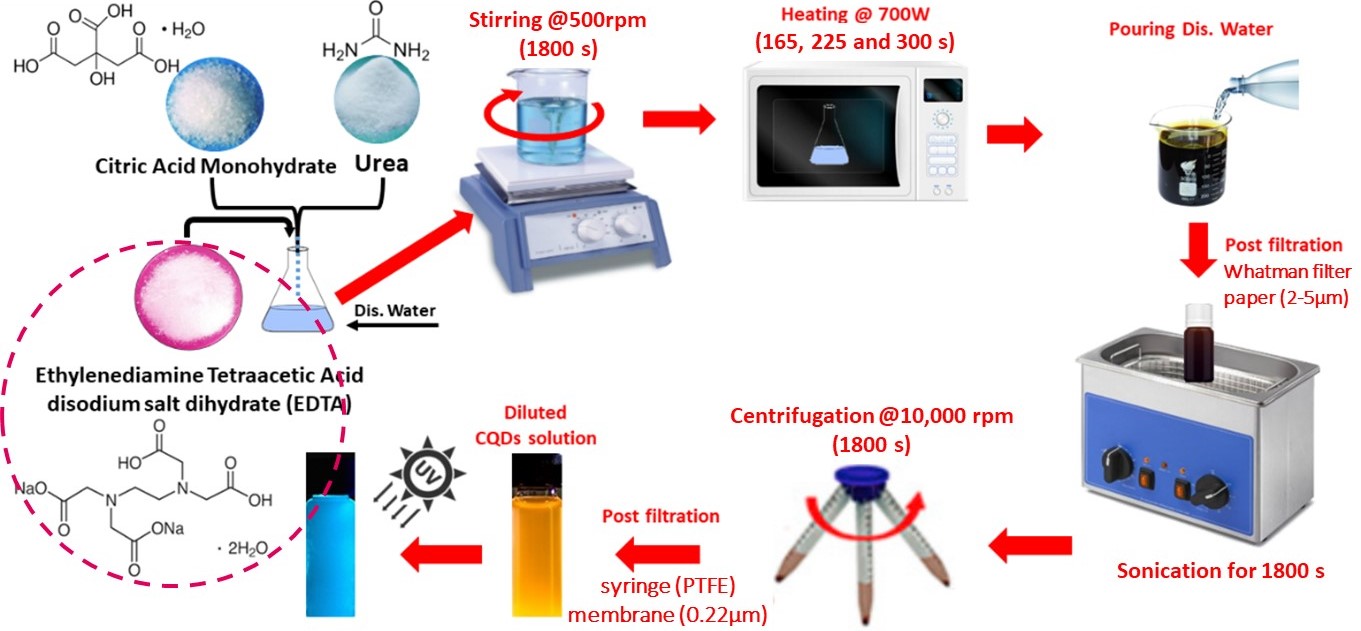
**

**Figure S1: Schematic of CQDs synthesis by Microwave irradiation**

**
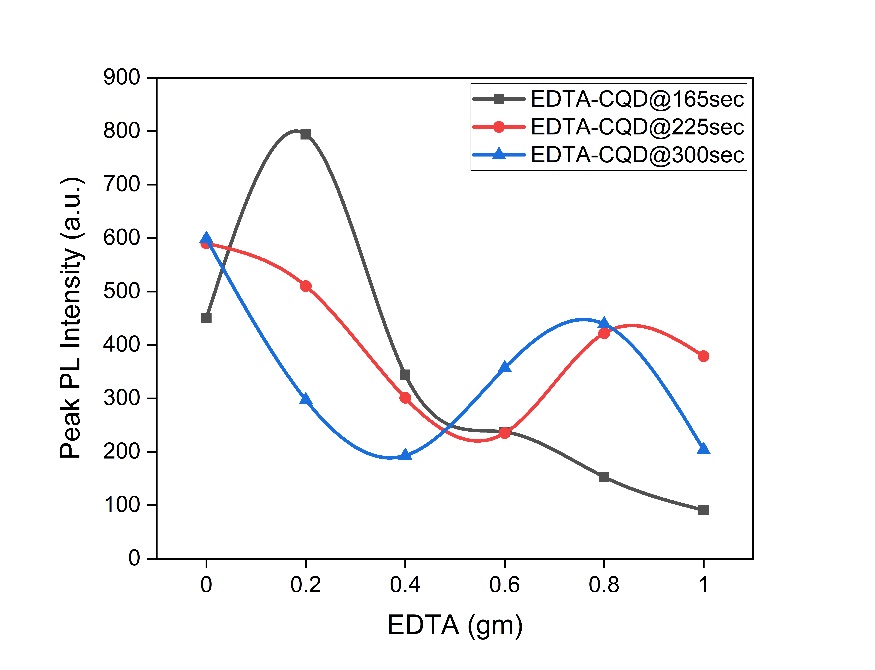
**

**Figure S2: Variation in Peak PL intensity of EDTA-CQD with EDTA concentration**


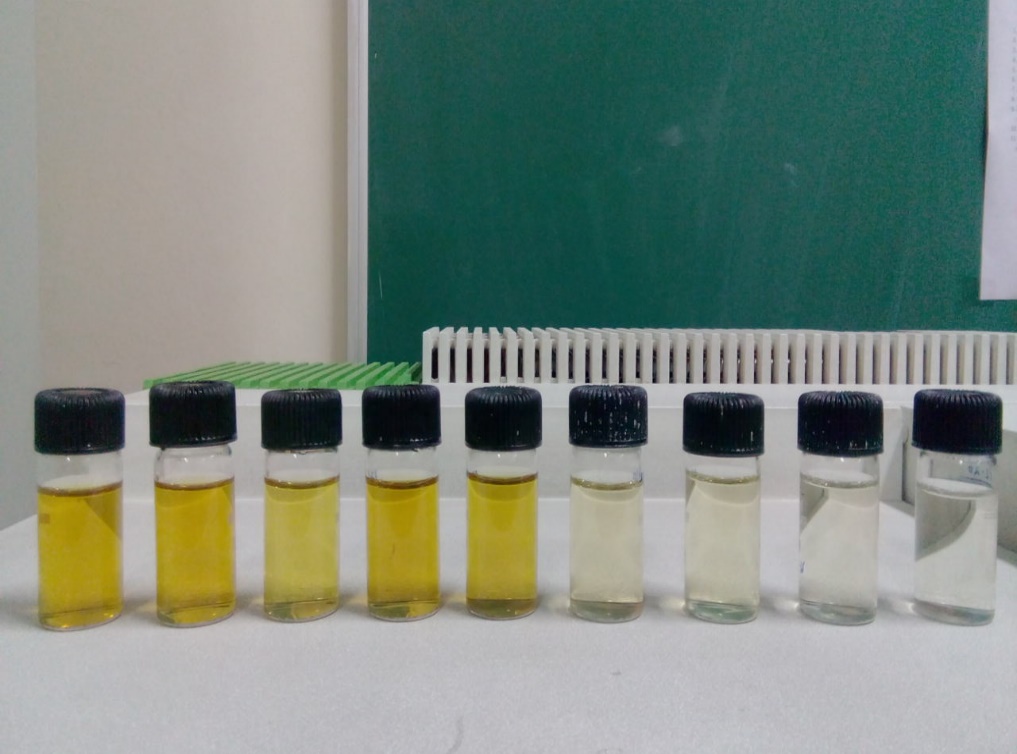

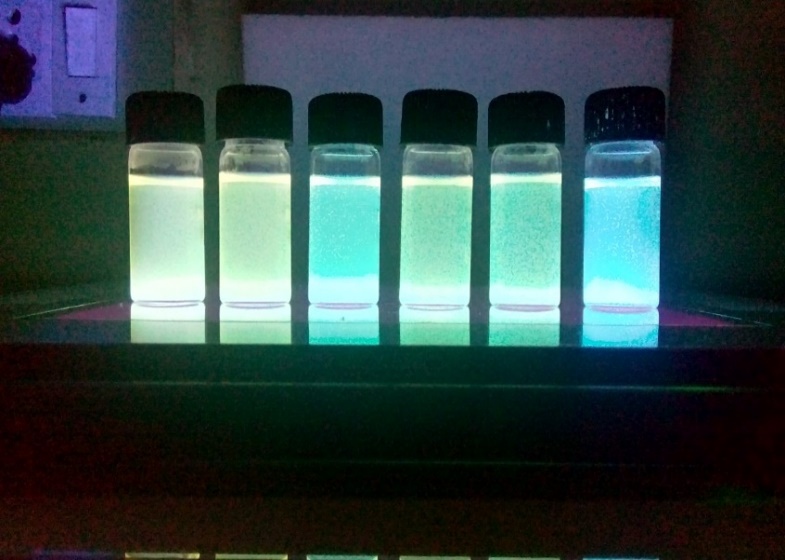


(a) (b)

**Figure S2 (a): EDTA-CQD@165sec-Left to Right (EDTA from 0.0g -1.0g in steps of 0.2g) exposed under (a) day light and (b) UV light**


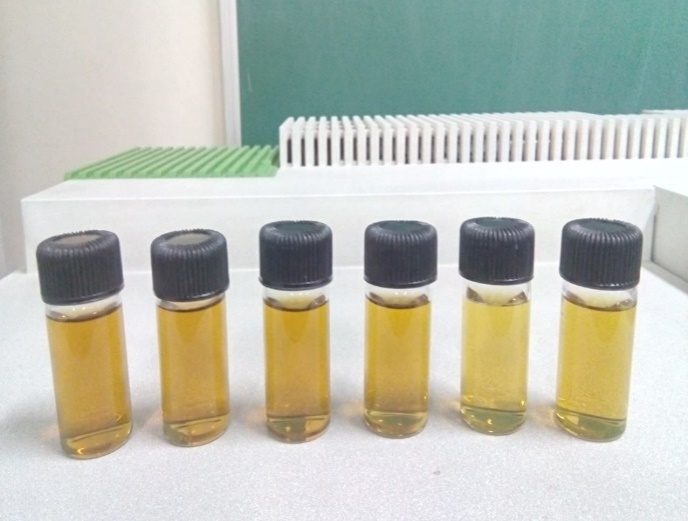

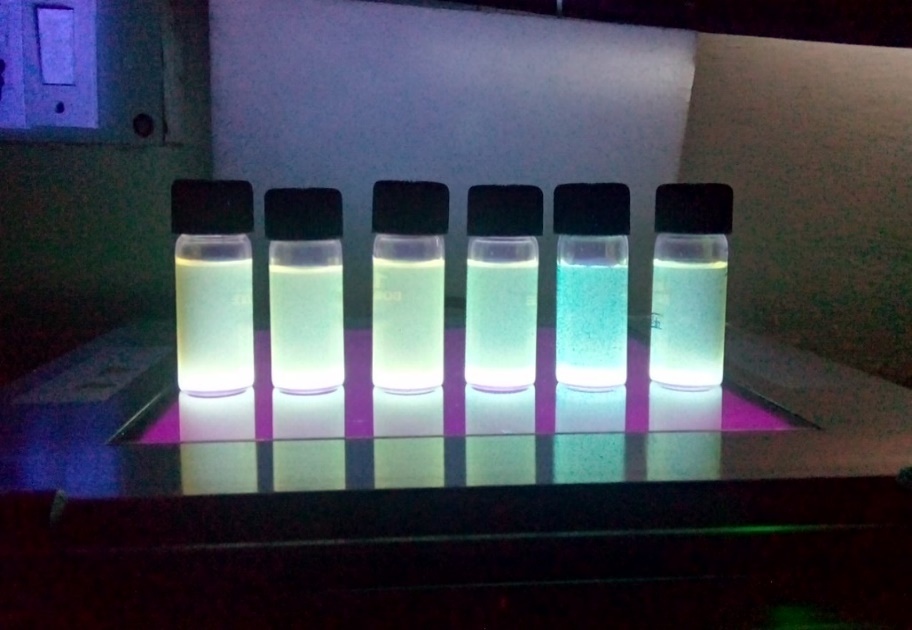


(a) (b)

**Figure S2 (b): EDTA-CQD@225sec-Left to Right (EDTA from 0.0g -1.0g in steps of 0.2g) exposed under (a) day light and (b) UV light**


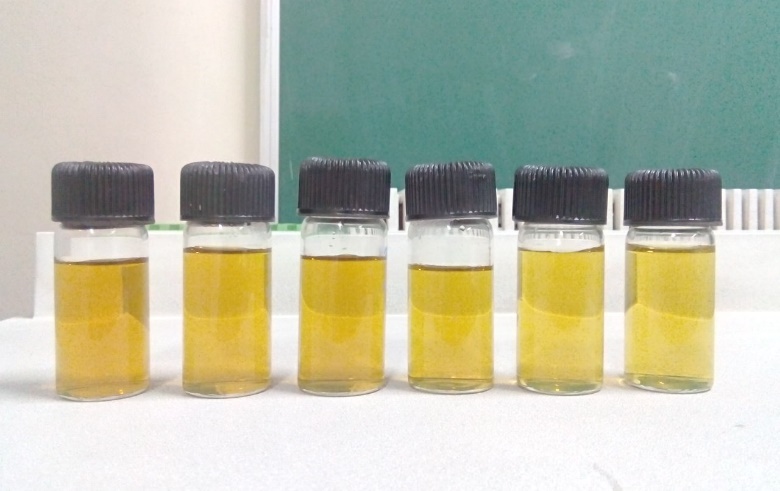

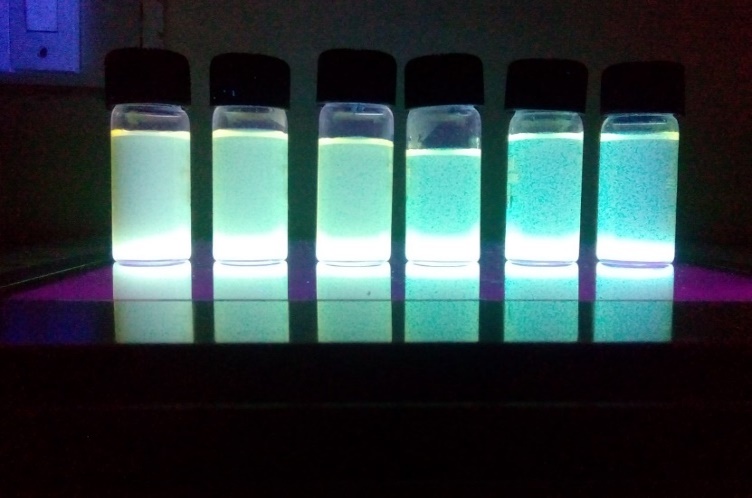


**(a) (b)**

**Figure S2 (c): EDTA-CQD@300sec-Left to Right (EDTA from 0.0g -1.0g in steps of 0.2g) exposed under (a) day light and (b) UV light**


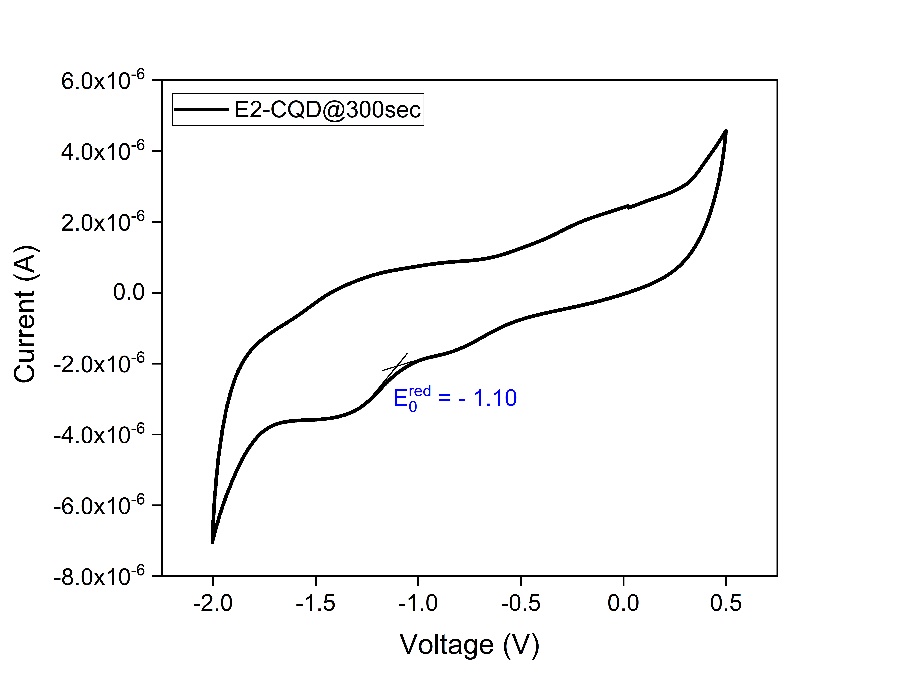


**Figure S3 (a): CV plot of E2-CQD@300sec**


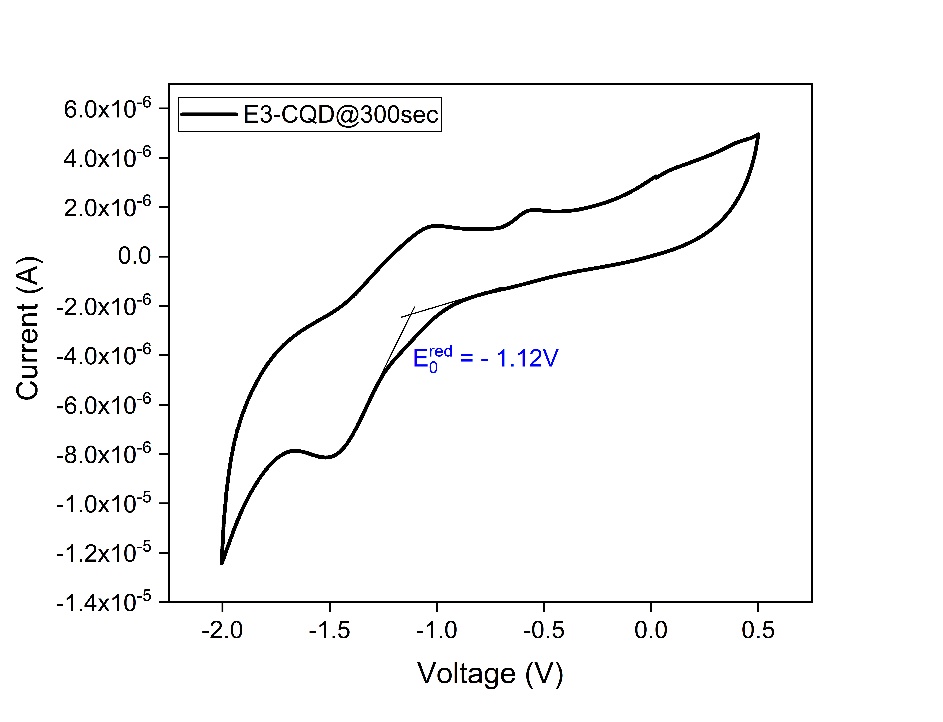


**Figure S3 (b): CV plot of E3-CQD@300sec**


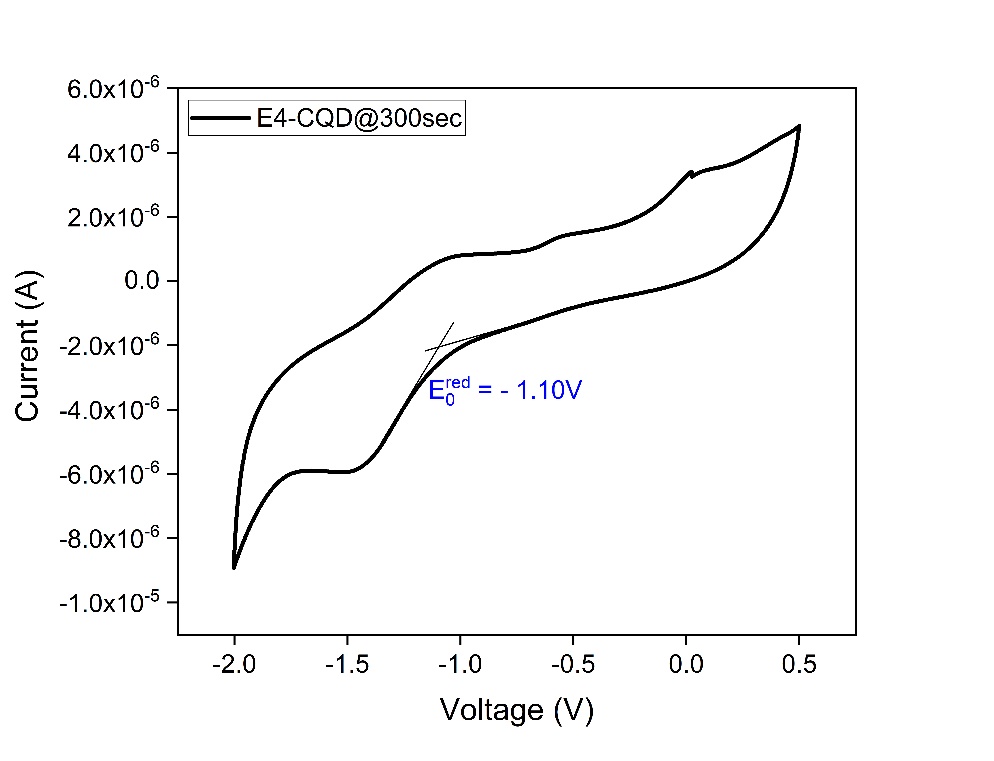


**Figure S3 (c): CV plot of E4-CQD@300sec**


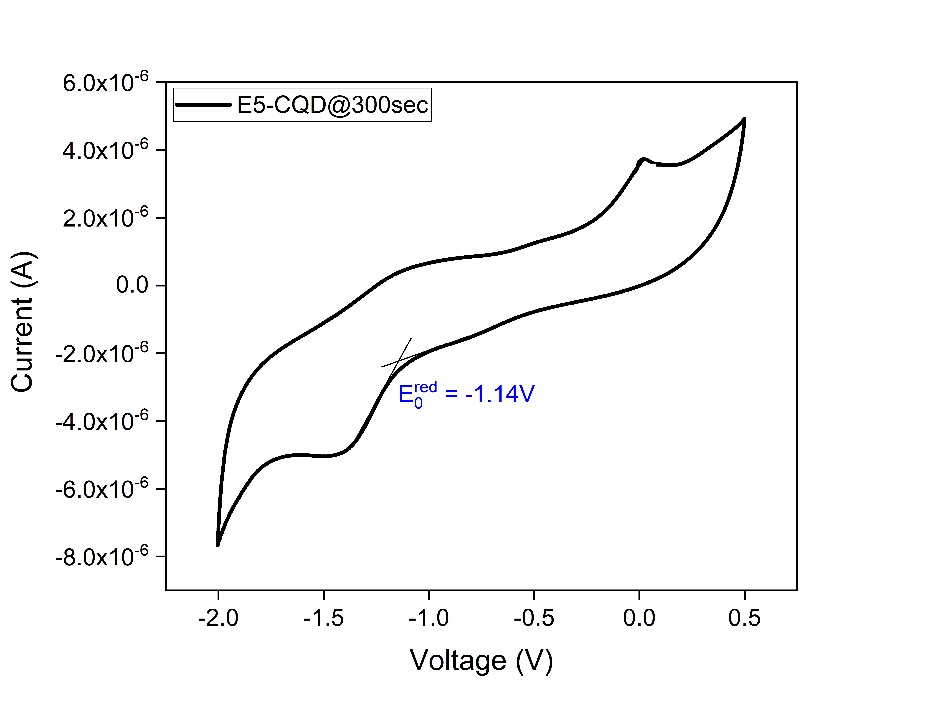


**Figure S3 (d): CV plot of E5-CQD@300sec**


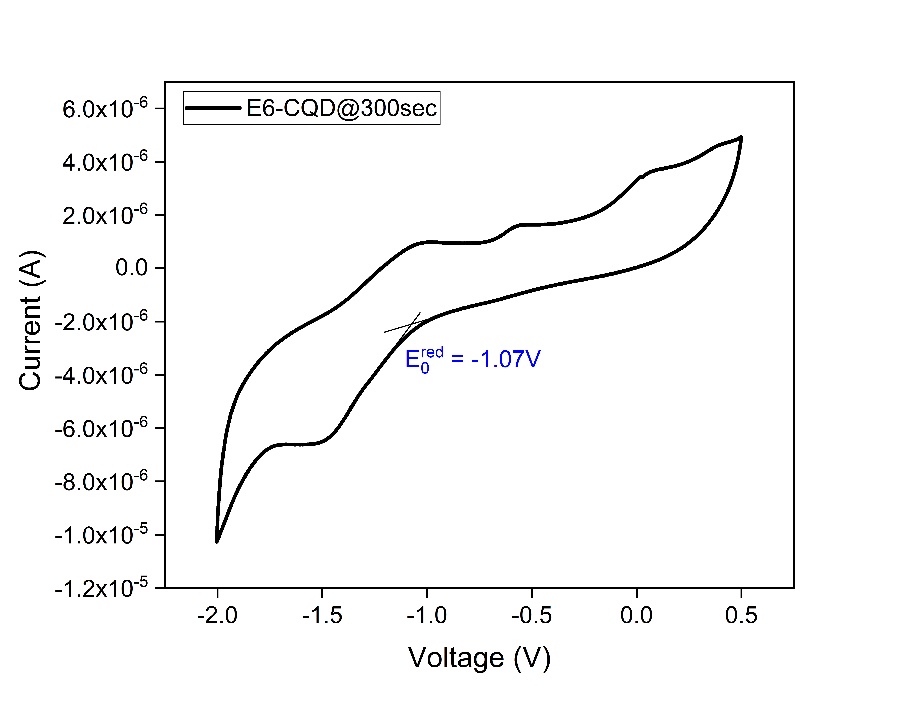


**Figure S3 (e): CV plot of E6-CQD@300sec**


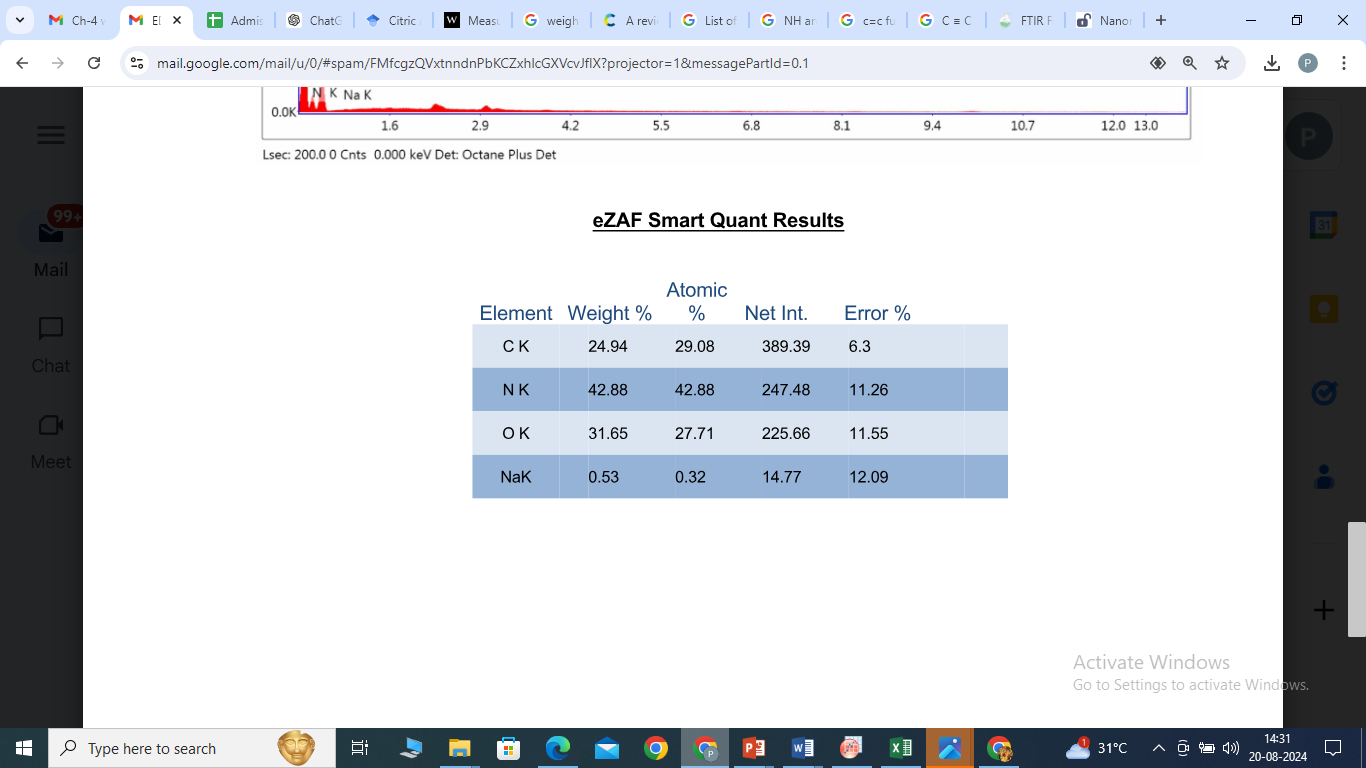

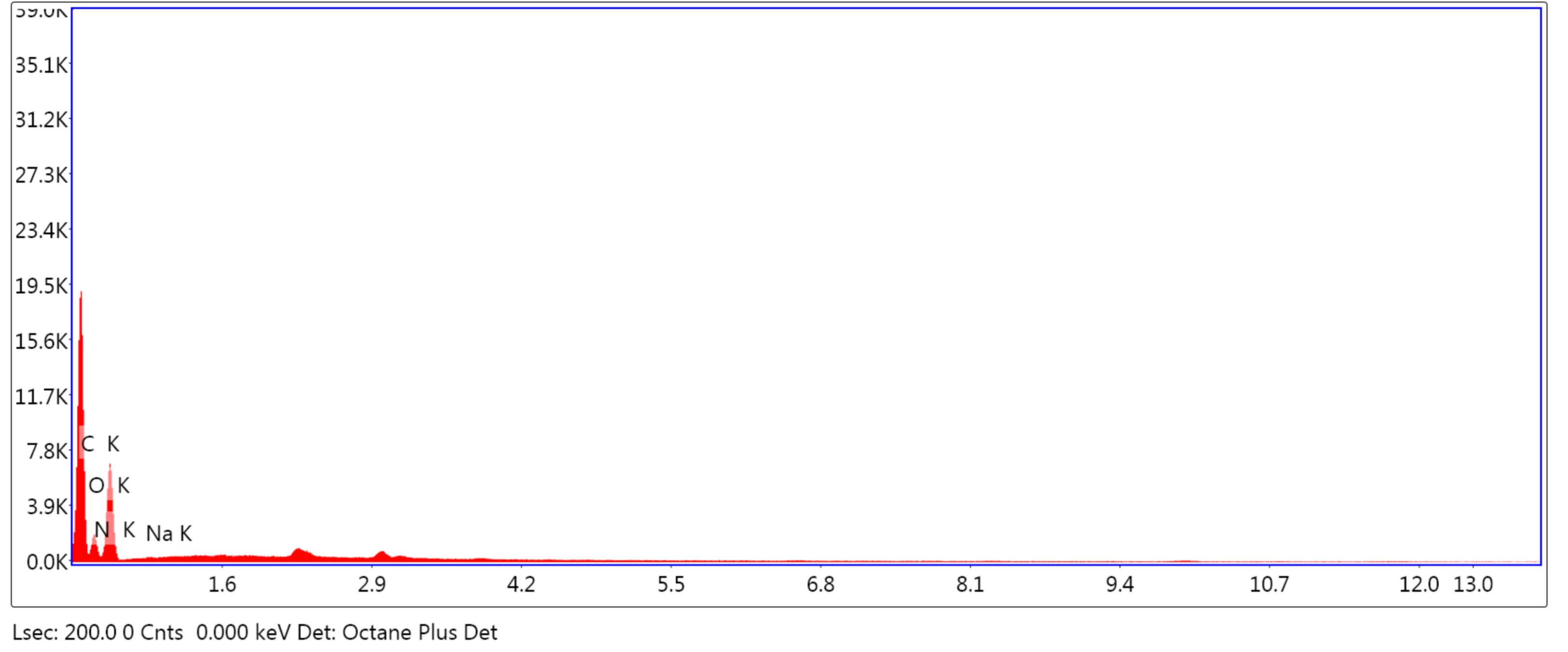


**Figure S4 (a): Peaks of elements presents in E1-CQD@300sec**


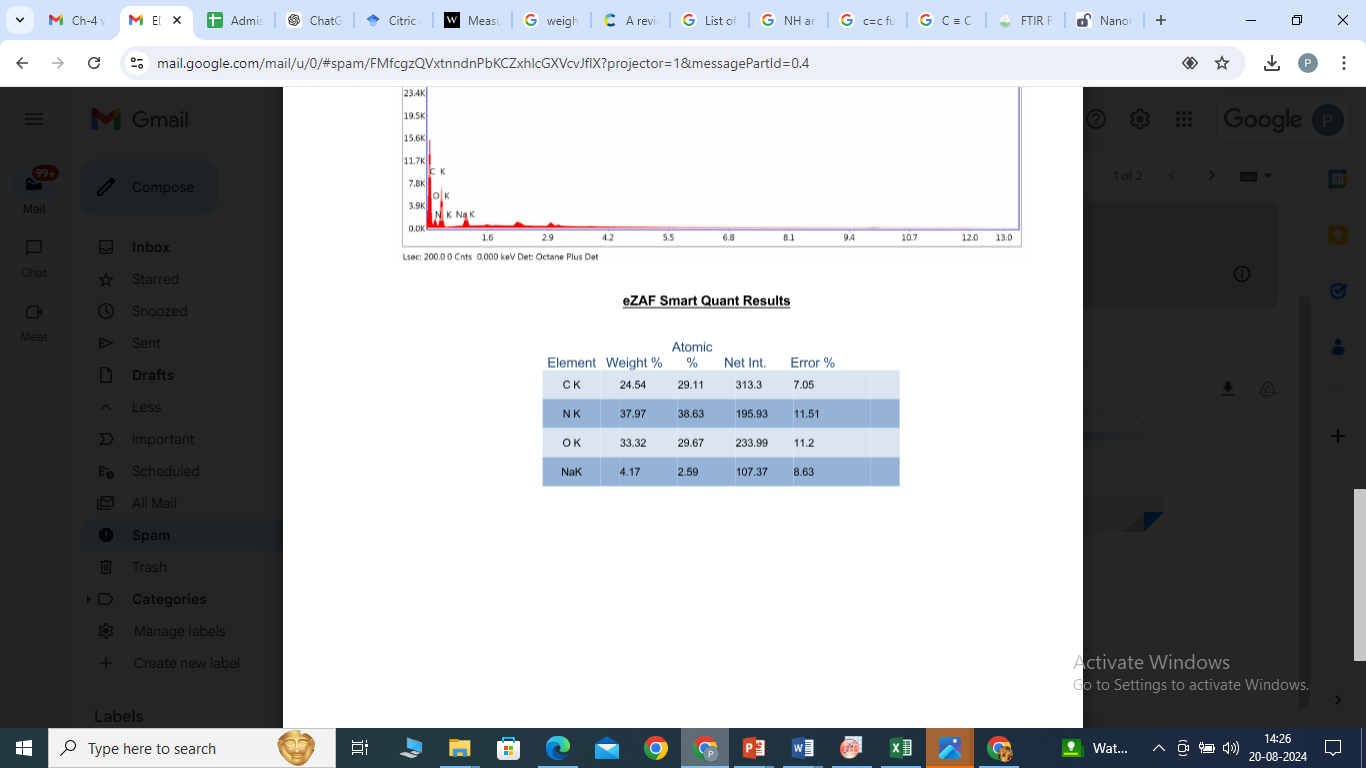

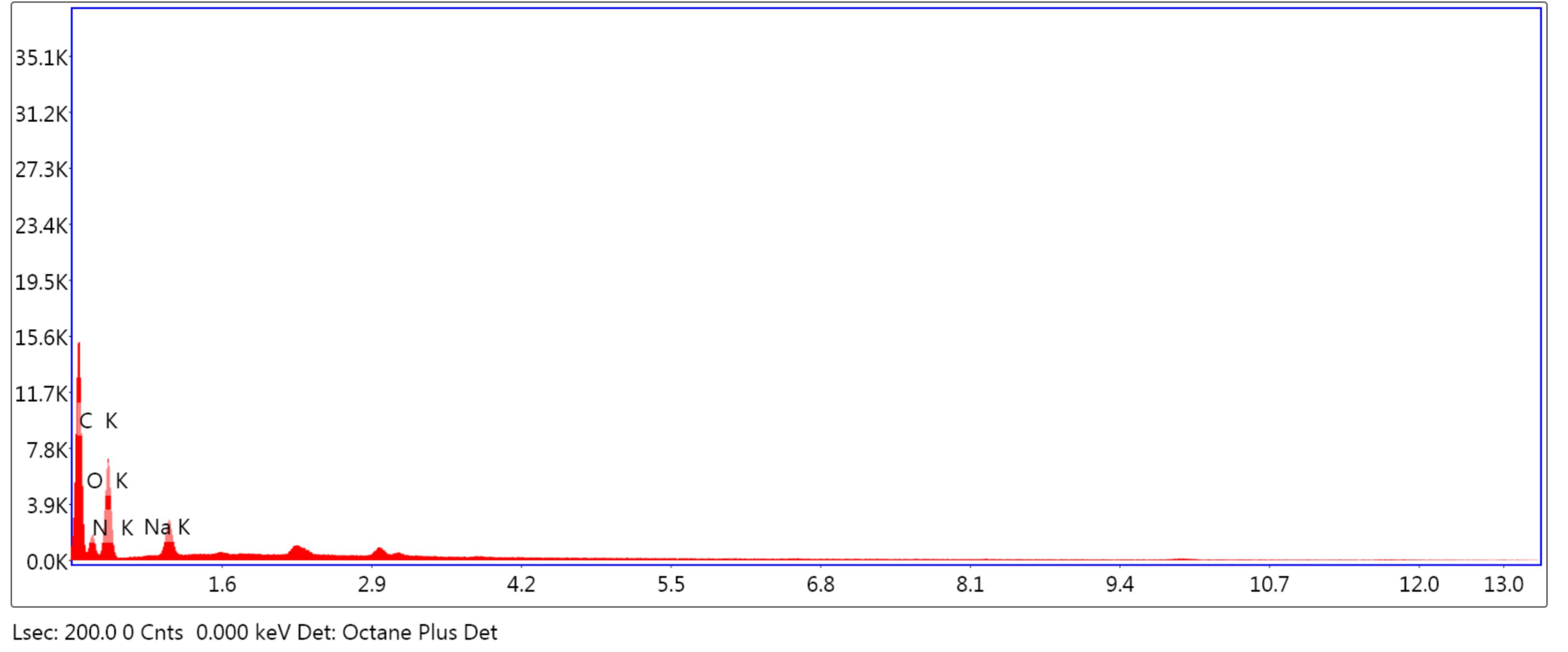


**Figure S4 (b): Peaks of elements presents in E2-CQD@300sec**


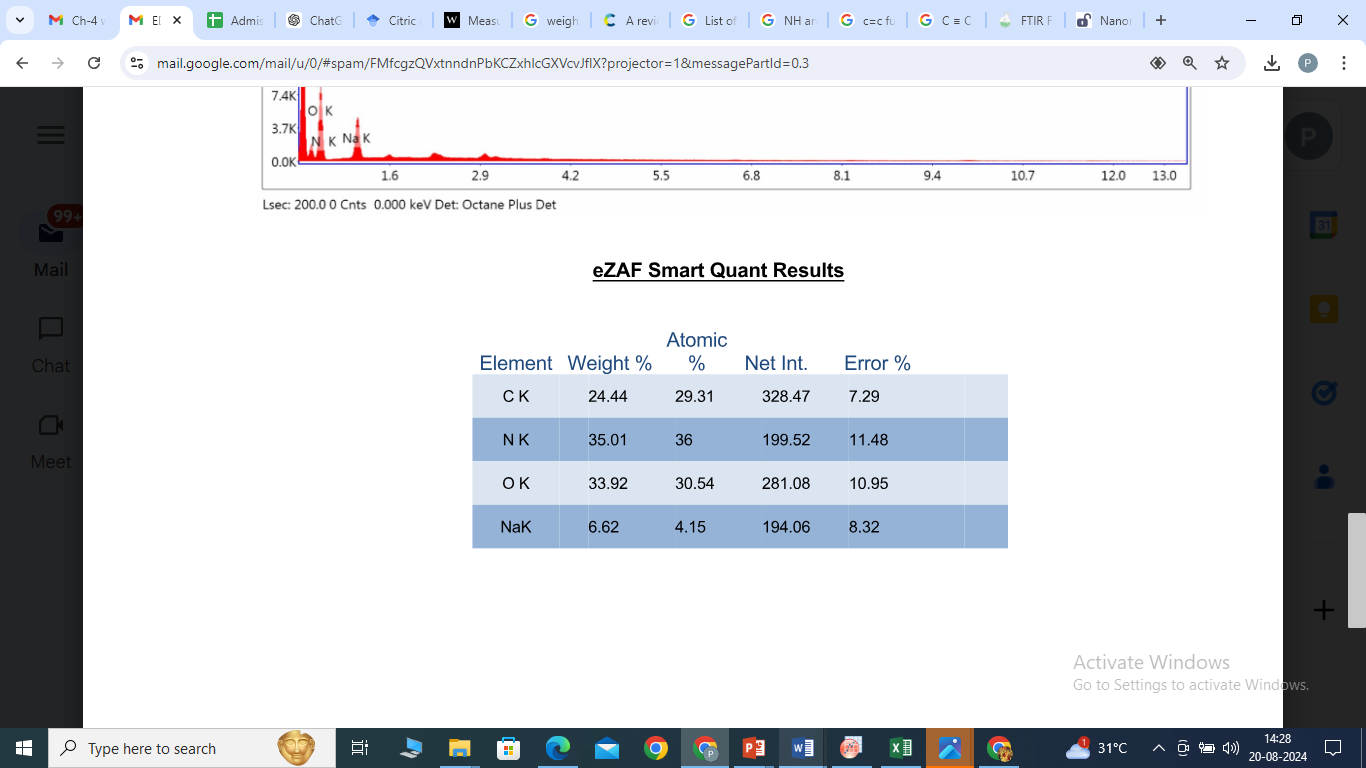

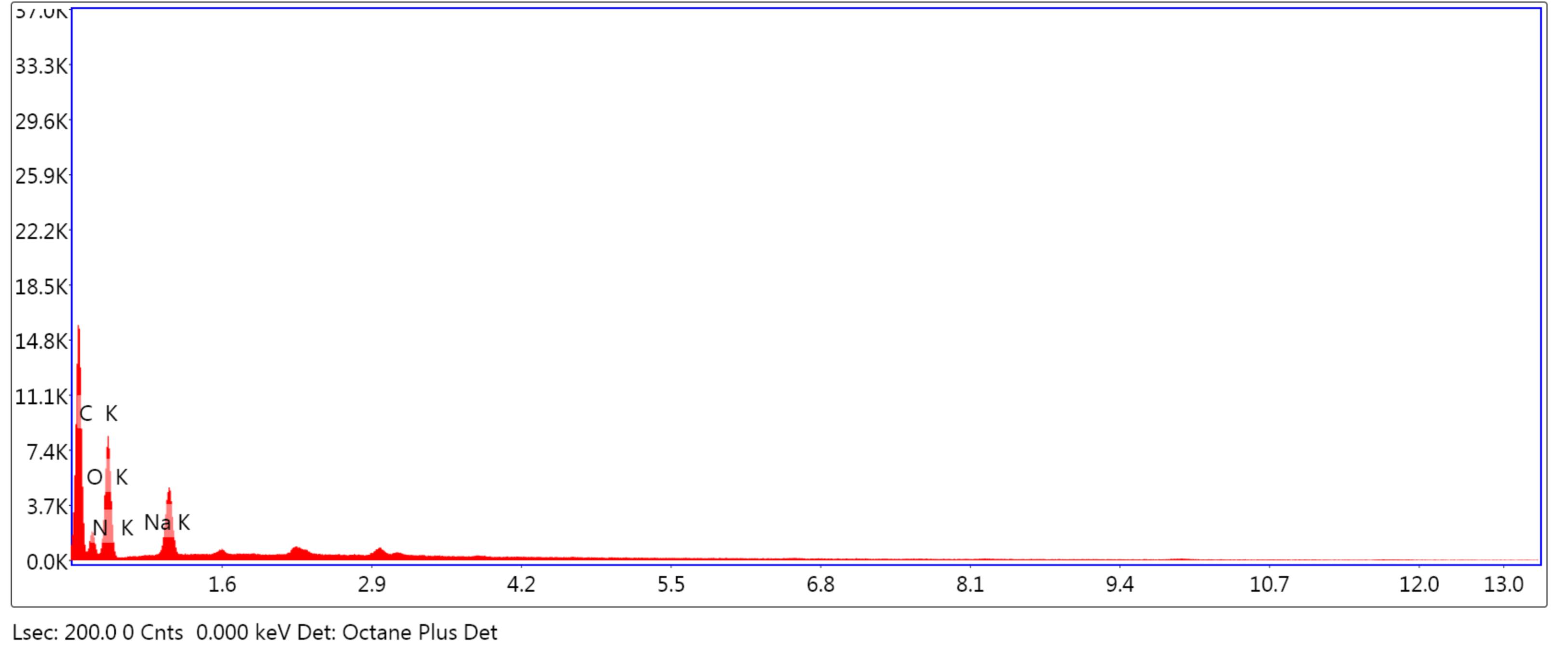


**Figure S4 (c): Peaks of elements presents in E3-CQD@300sec**


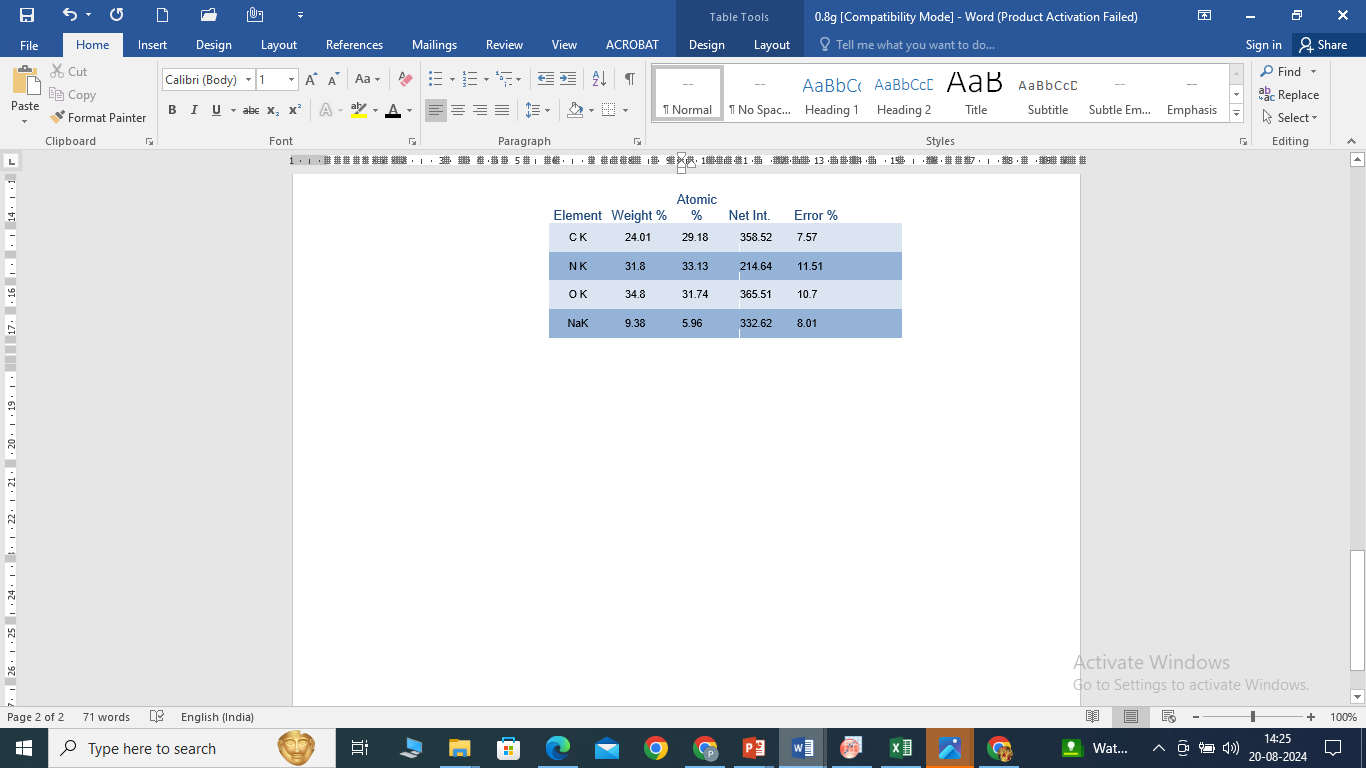

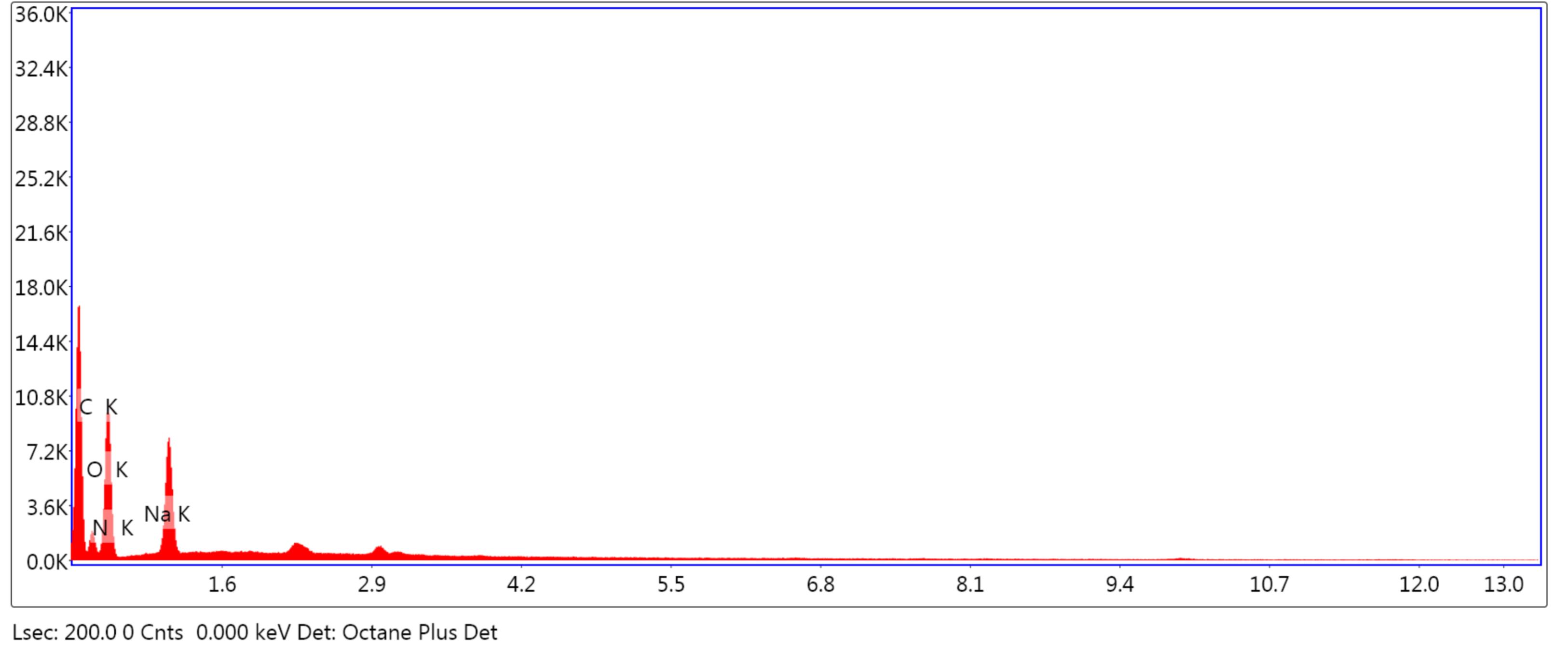


**Figure S4 (d): Peaks of elements presents in E5-CQD@300sec**


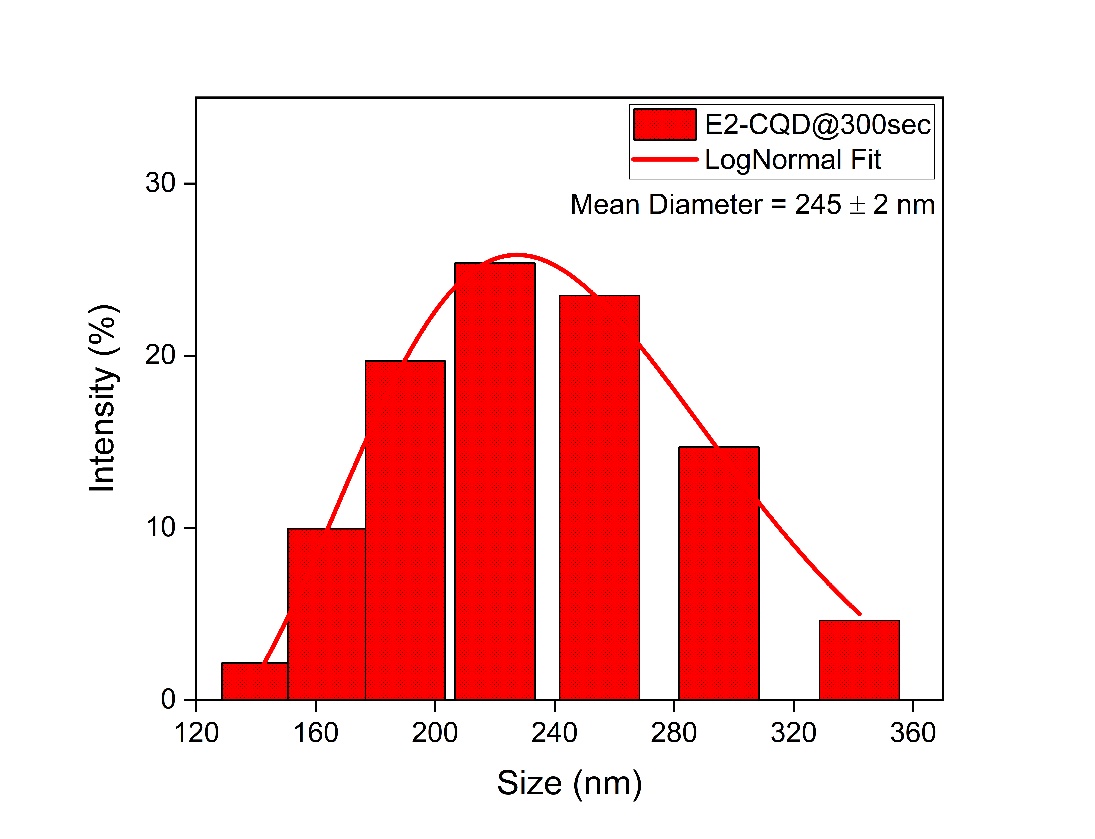


**Figure S5 (a): Size distribution of E2-CQD@300sec**


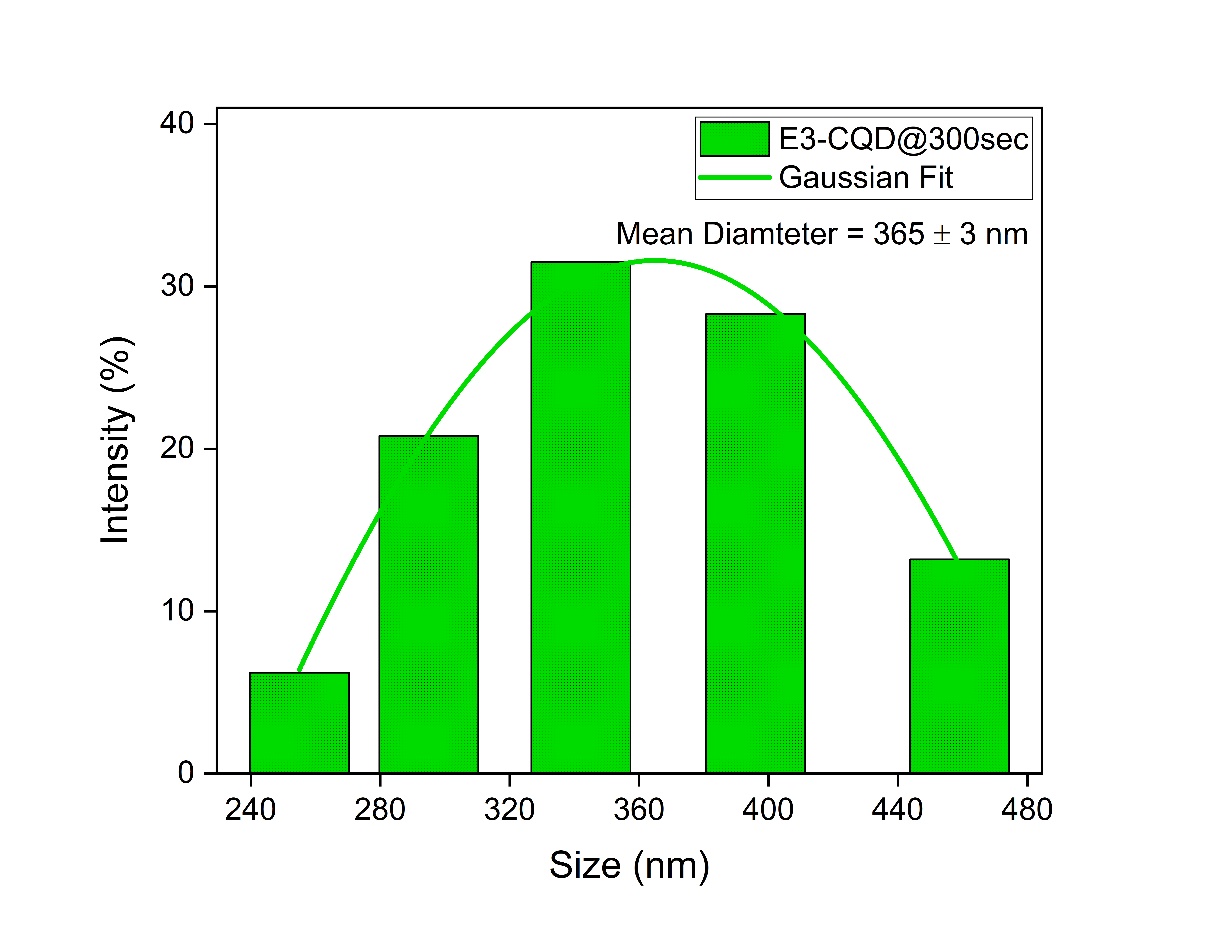


**Figure S5 (b): Size distribution of E3-CQD@300sec**


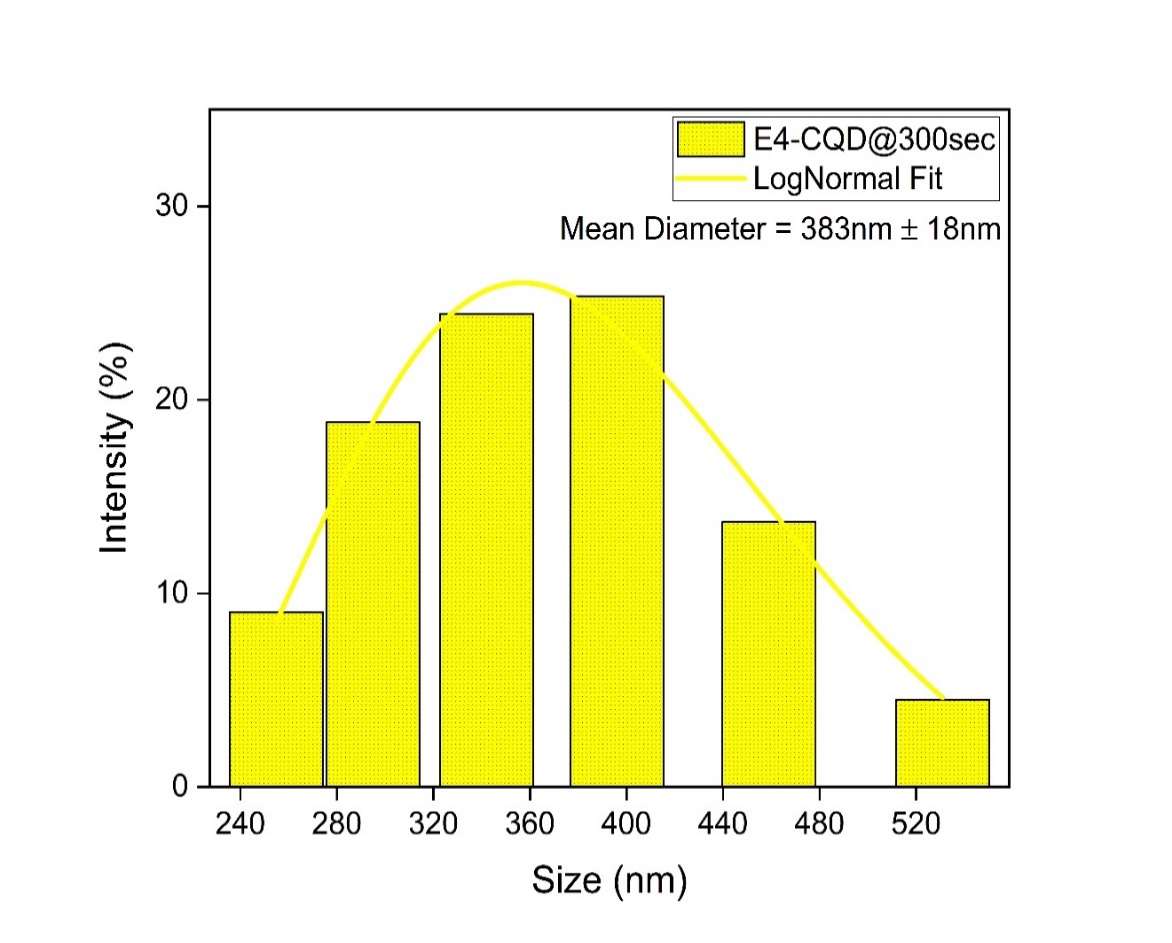


**Figure S5 (c): Size distribution of E4-CQD@300sec**


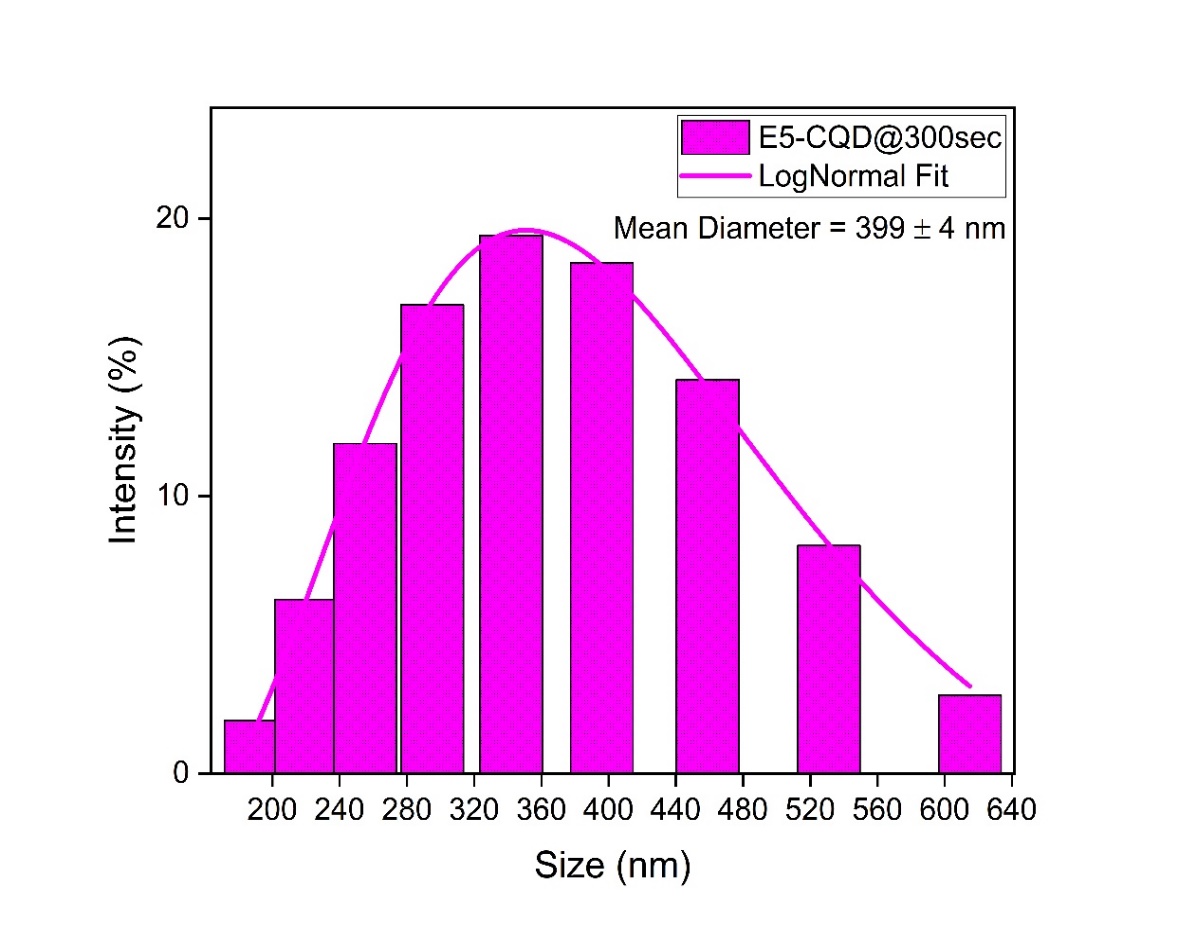


**Figure S5 (d): Size distribution of E5-CQD@300sec**


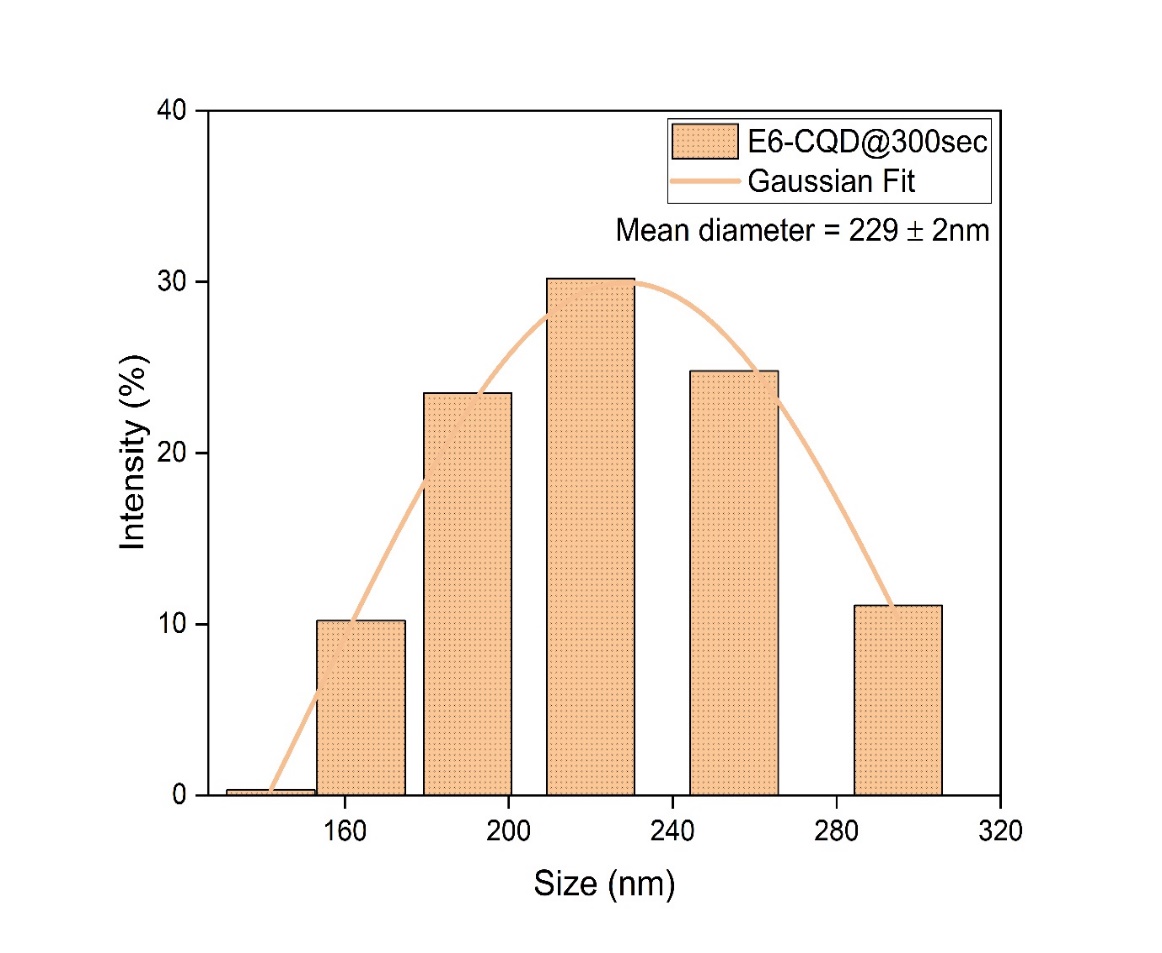


**Figure S5 (e): Size distribution of E6-CQD@300sec**


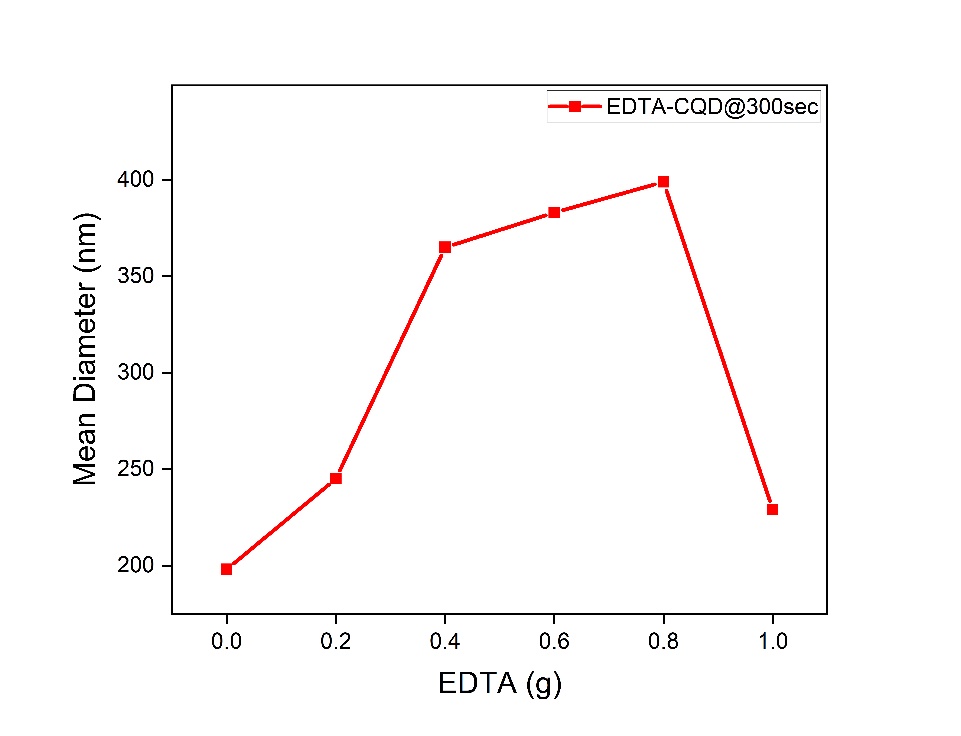


**Figure S6: Size variation of EDTA-CQD@300sec with EDTA variation**


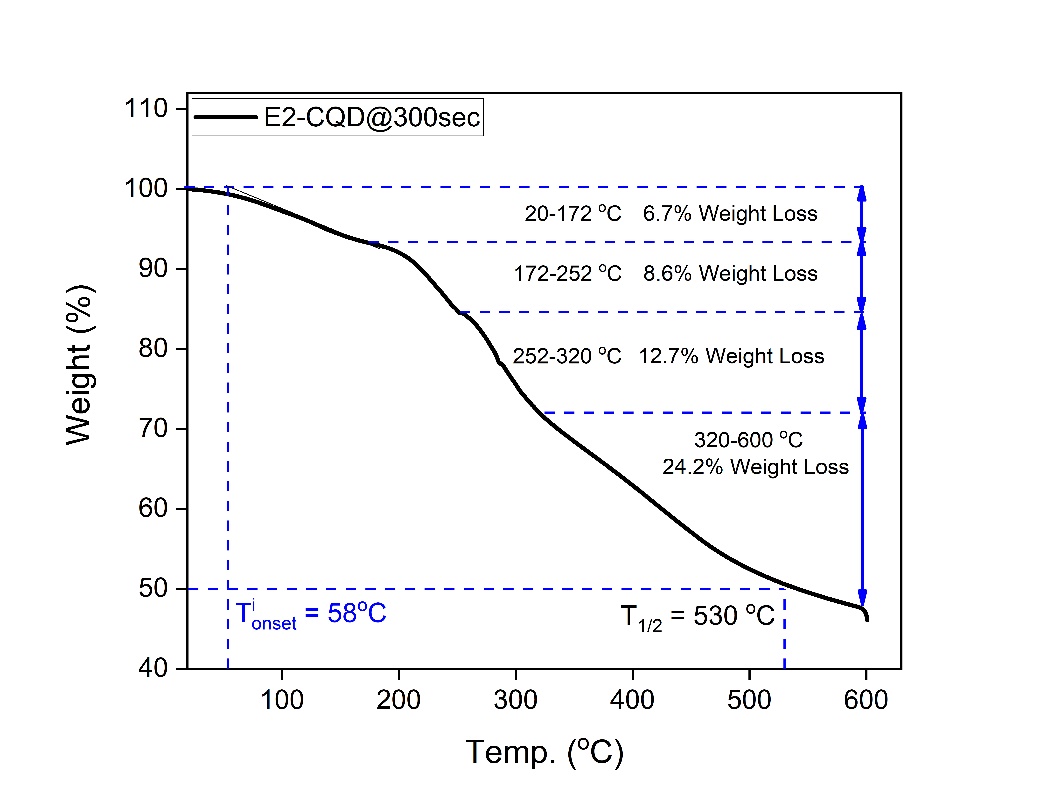


**Figure S7 (a): Thermal stability analysis of E2-CQD@300sec**


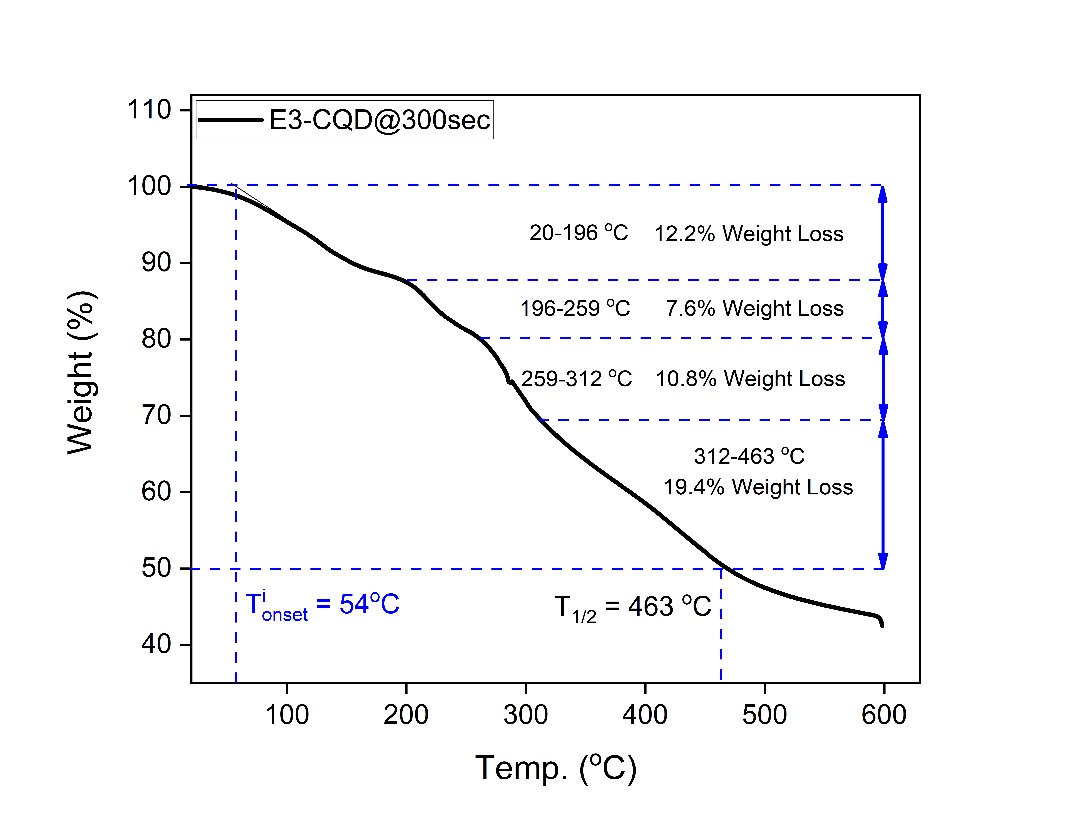


**Figure S7 (b): Thermal stability analysis of E3-CQD@300sec**


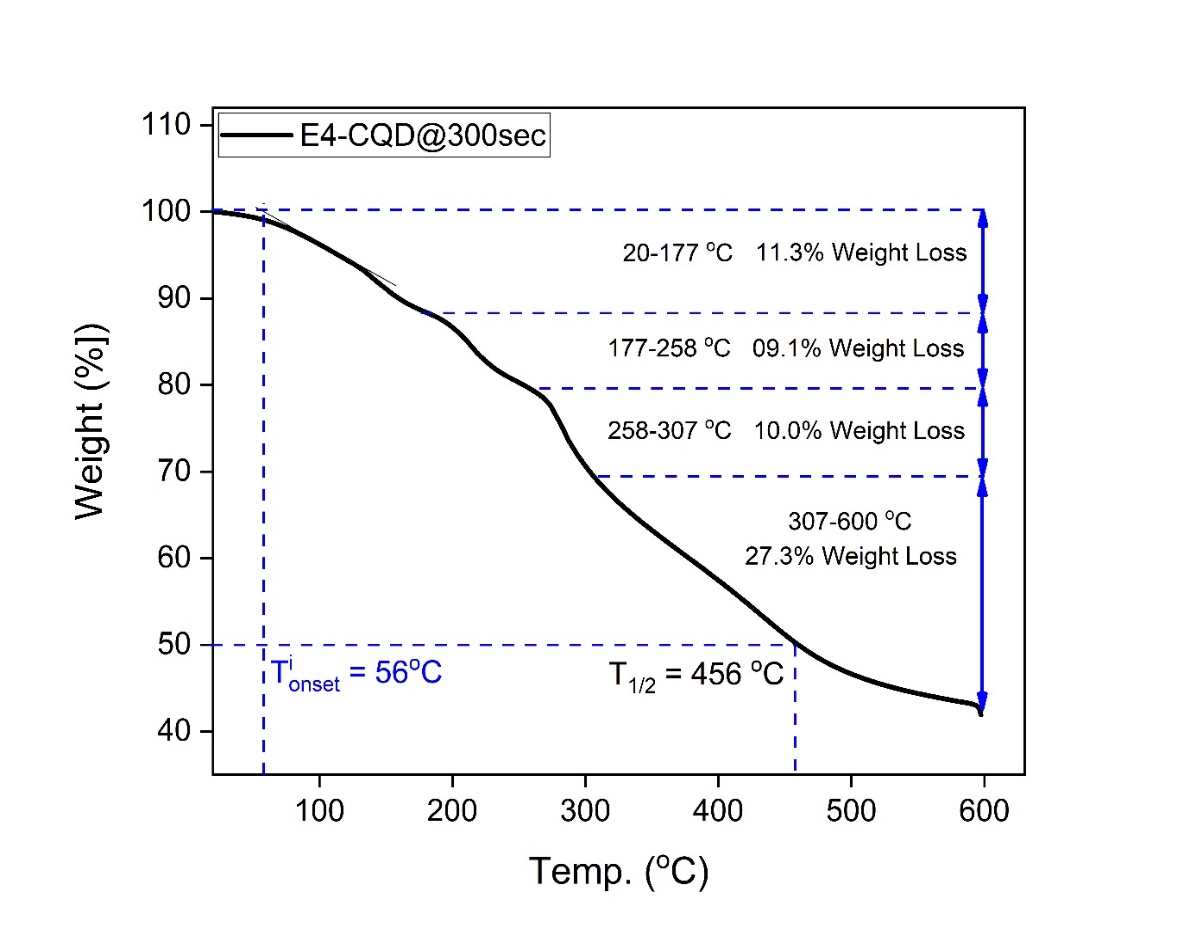


**Figure S7 (c): Thermal stability analysis of E4-CQD@300sec**


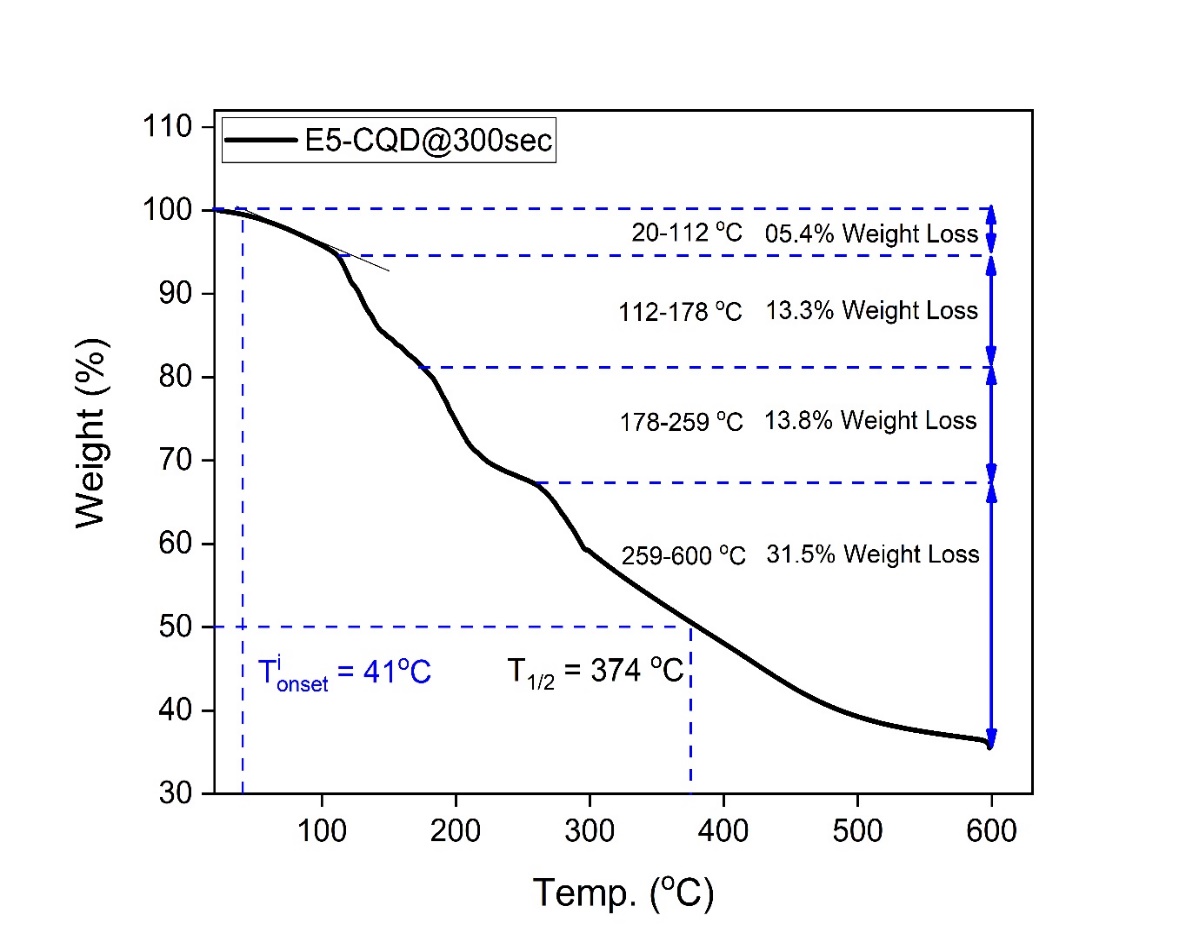


**Figure S7 (d): Thermal stability analysis of E5-CQD@300sec**


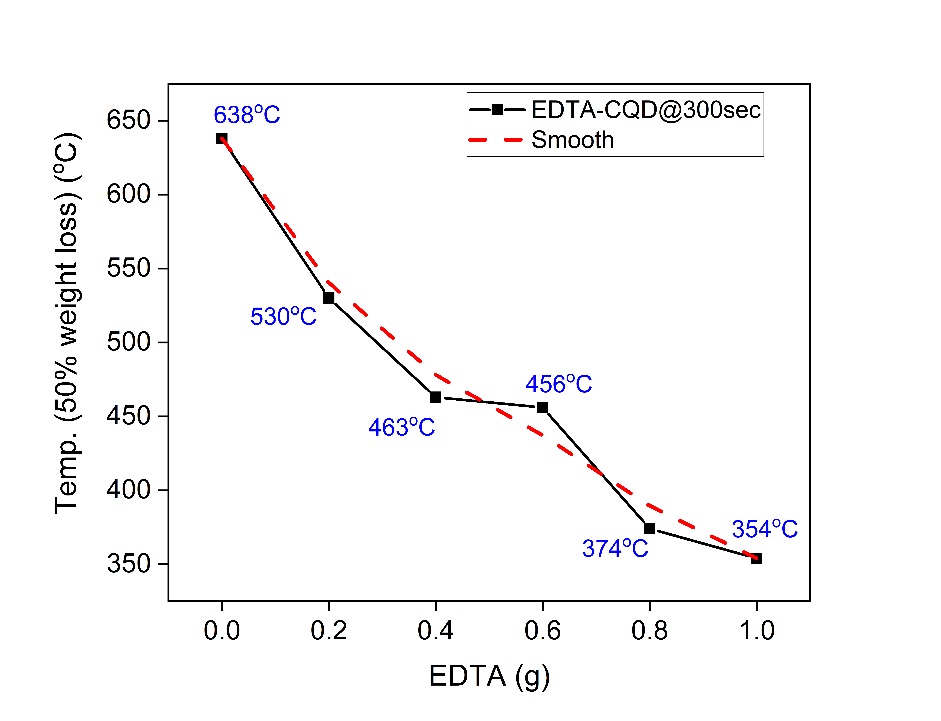


**Figure S8: 50% weight loss of EDTA-CQD@300sec for varying concentration of EDTA**

**Table S1: The Net absorbance of different CQD-EDTA by evaluating the area of absorbance curves over the range of 300-650nm**

| **EDTA (gm)** | **Net Absorbance (a.u.)** | **Net Absorbance (a.u.)** | **Net Absorbance (a.u.)** |
| --- | --- | --- | --- |
|  | **EDTA-CQD@165sec** | **EDTA-CQD@225sec** | **EDTA-CQD@300sec** |
| 0.0 | 10.91 | 13.41 | 13.65 |
| 0.2 | 11.74 | 12.2 | 12.7 |
| 0.4 | 7.06 | 12.04 | 10.89 |
| 0.6 | 11.61 | 10.83 | 11.8 |
| 0.8 | 9.77 | 8.26 | 10.66 |
| 1.0 | 4.91 | 8.99 | 10.42 |

**Table S2: Variation of crystallite size with EDTA concentration**

| **Sample** | | **EDTA**  **(gm)** | **FWHM**  **(radians)** | **2θ**  **(degree)** | **Cos(θ)**  **(radians)** | **Crystallite Size**  **(nm)** |
| --- | --- | --- | --- | --- | --- | --- |
| EDTA-CQD@300sec | E1-CQD@300sec | 0.0 | 0.276 | 26.451 | 0.973 | 0.517 |
|  | E2-CQD@300sec | 0.2 | 0.354 | 26.141 | 0.974 | 0.402 |
|  | E3-CQD@300sec | 0.4 | 0.379 | 25.541 | 0.976 | 0.376 |
|  | E4-CQD@300sec | 0.6 | 0.400 | 25.100 | 0.976 | 0.355 |
|  | E5-CQD@300sec | 0.8 | 0.418 | 24.972 | 0.976 | 0.340 |
|  | E6-CQD@300sec | 1.0 | 0.423 | 24.851 | 0.976 | 0.336 |
